# Activation and Alliance of Regulatory Pathways in *C. albicans* during Mammalian Infection

**DOI:** 10.1371/journal.pbio.1002076

**Published:** 2015-02-18

**Authors:** Wenjie Xu, Norma V. Solis, Rachel L. Ehrlich, Carol A. Woolford, Scott G. Filler, Aaron P. Mitchell

**Affiliations:** 1 Department of Biological Sciences, Carnegie Mellon University, Pittsburgh, Pennsylvania, United States of America; 2 Division of Infectious Diseases, Los Angeles Biomedical Research Institute at Harbor-UCLA Medical Center, Torrance, California, United States of America; University of Aberdeen, UNITED KINGDOM

## Abstract

Gene expression dynamics have provided foundational insight into almost all biological processes. Here, we analyze expression of environmentally responsive genes and transcription factor genes to infer signals and pathways that drive pathogen gene regulation during invasive *Candida albicans* infection of a mammalian host. Environmentally responsive gene expression shows that there are early and late phases of infection. The early phase includes induction of zinc and iron limitation genes, genes that respond to transcription factor Rim101, and genes characteristic of invasive hyphal cells. The late phase includes responses related to phagocytosis by macrophages. Transcription factor gene expression also reflects early and late phases. Transcription factor genes that are required for virulence or proliferation in vivo are enriched among highly expressed transcription factor genes. Mutants defective in six transcription factor genes, three previously studied in detail (Rim101, Efg1, Zap1) and three less extensively studied (Rob1, Rpn4, Sut1), are profiled during infection. Most of these mutants have distinct gene expression profiles during infection as compared to in vitro growth. Infection profiles suggest that Sut1 acts in the same pathway as Zap1, and we verify that functional relationship with the finding that overexpression of either *ZAP1* or the Zap1-dependent zinc transporter gene *ZRT2* restores pathogenicity to a *sut1* mutant. Perturbation with the cell wall inhibitor caspofungin also has distinct gene expression impact in vivo and in vitro. Unexpectedly, caspofungin induces many of the same genes that are repressed early during infection, a phenomenon that we suggest may contribute to drug efficacy. The pathogen response circuitry is tailored uniquely during infection, with many relevant regulatory relationships that are not evident during growth in vitro. Our findings support the principle that virulence is a property that is manifested only in the distinct environment in which host–pathogen interaction occurs.

## Abstract 2

A study of the invasive infection of a mammalian host by the pathogenic fungus *Candida albicans* reveals characteristic gene regulation patterns in response to the host environment, distinct from those seen when growing in vitro.

## Author Summary

We have a limited understanding of how the expression of pathogens’ genes changes during infection of humans or other animal hosts, in contrast to *in vitro* models of infection. Here we profile the alteration in gene expression over time as a predictor of functional consequences during invasive growth of *Candida* in the kidney; a situation in which the limited number of pathogen cells makes gene expression challenging to assay. Our findings reveal that there are distinct early and late phases of infection, and identify new genes that govern the early zinc acquisition response necessary for proliferation *in vivo*—and thus required for infection. We also find that the response to drug treatment that manifests during infection can be distinct from that detected *in vitro*. We show that a well-known gene expression response to the antifungal drug caspofungin is naturally down-regulated in infecting cells, suggesting that the efficacy of the drug may be enhanced by a susceptible state of the pathogen during invasive proliferation.

## Introduction

Which genes does a pathogen express during infection? Which regulatory pathways govern expression of those genes in vivo? These questions are central to the study of microbial pathogenesis, and they have been addressed by diverse approaches [1–4]. Despite those efforts, we have a limited understanding of gene expression dynamics during infection of humans or animals by most pathogens. Even new genome-wide technologies on the horizon face several well-acknowledged technical hurdles before they can be implemented in tissues of infected animals [5]. Here, we have used an exquisitely sensitive technology to elucidate gene regulation during tissue invasion by the fungal pathogen *Candida albicans*.

*C*. *albicans* is a human commensal that lives on mucosal surfaces of the gastrointestinal and genitourinary tracts [6]. Deep tissue infection begins when the organism gains access to the bloodstream due to disruption of mucosal surfaces or biofilm growth on an implanted device. *C*. *albicans* disseminates via the bloodstream and can infect almost any tissue [6]. A mouse hematogenously disseminated candidiasis (HDC) infection model, in which *C*. *albicans* yeast cells are inoculated into the lateral tail vein, has been widely used to study invasive candidiasis [7]. Although *C*. *albicans* invades and infects virtually all tissues, the kidney is the principal target organ. In the kidney, *C*. *albicans* proliferates as hyphae [7], which are long tubular cells attached end to end. During the first 12 hr postinfection, relatively few fungal cells are present in the kidney. Pro-inflammatory cytokines, including TNFα and IL-1β, are detected in the kidney and in circulation by this time [7]. By 24 hr, the fungal burden increases by a factor of 100, and leukocyte infiltration begins. By 48 hr, the fungal burden increases by another factor of 10, and leukocyte infiltration is extensive [7].

Prior studies have profiled gene expression in the kidney during invasive *C*. *albicans* infection using microarray technology [4,8,9]. These pioneering studies established several basic principles that have shaped the thinking in *Candida* infection biology. Specifically, examination of *C*. *albicans* gene expression revealed the induction of stress response genes, adhesins, and fatty acid utilization genes during infection [4,8]. One study, which used yeast-form cell RNA for comparison, detected induction of hyphal genes, as expected from the extensive hyphal growth observed in infected kidney [8]. These findings argued that adaptation ability is central for proliferation in a novel niche like the kidney, and that hyphal morphogenesis during infection is accompanied by the hyphal gene expression program that has been characterized during growth in vitro. Host gene expression in the kidney is also broadly affected at 24 hr postinfection [9]. There is extensive induction of pro-inflammatory cytokine genes, including IL-6, Kc/Cxcl1, Cxcl10, Cxcl11, Cxcl13, IL-1β, and Tnfα [9]. In addition, there is substantial induction of host pattern recognition receptor genes and their signaling pathway components, including TLR2, Dectin-2,DC-Sign, and Myd88 [9]. These profiling studies paint a picture of kidney invasion in which *C*. *albicans* proliferates in hyphal form, adapting to such stresses as the need to use alternative carbon sources, and induces a pro-inflammatory response in the host that ultimately leads to leukocyte trafficking into the kidney.

While prior profiling studies have provided foundational insight into mammalian infection, the dynamic range and sensitivity of microarray signals [5,10,11] may have limited detection of key regulatory genes that govern the infection process. In addition, while reliable gene expression signals were recorded for infection with wild-type *C*. *albicans* strains, it was unclear whether those profiling approaches could be applied under conditions in which organ fungal burden was diminished, such as with attenuated mutants or after drug therapy. We have recently implemented nanoString technology for analysis of pathogen gene expression during infection [12,13]. For oral and abdominal *C*. *albicans* infection models, in which pathogen cells are relatively numerous, we have been able to collect a snapshot of infection samples and elucidate the roles of two well-established virulence regulators, Bcr1 and Rim101 [12,13]. Here we turn our attention to the most widely used *C*. *albicans* infection model to capture the first time-course analysis, to our knowledge, of large-scale gene expression during invasive *Candida* infection. Our findings elucidate the unique functional interactions among virulence regulators that are manifested during infection and document an unexpected relationship between genes that respond to the infection environment and to antifungal therapy.

## Results

### Environmentally Responsive Gene Expression during Invasive Infection

We used a nanoString n-counter [10] to quantify selected *C*. *albicans* gene transcripts in whole kidney lysates at 12, 24, and 48 hr postinfection. We first tested our methodology using a probe set that contained 248 environmentally responsive genes chosen from previously described genome-wide datasets (S1 Data). Expression levels of these genes are known to respond to a range of signals, such as nutrient limitation and cell morphogenesis. Also, many of the response pathways that govern their expression are known to impact virulence, as inferred from mutant analysis and comparisons to other pathogens [14]. NanoString probe background signals were assessed by comparing uninfected and infected tissue samples. Signal-to-noise ratios for the majority of probes in all samples fell into the range of 10^1^ to 10^6^ (S1 Fig.). Agreement among independent samples was excellent (S1 Fig.; R^2^ values of 0.94 to >0.99), and induction ratios were confirmed by quantitative reverse transcription polymerase chain reaction (QRT-PCR) for all eight genes examined (S2 Fig.). Probe signals from infected tissue samples varied over a range of three orders of magnitude, and comparison with pure *C*. *albicans* cultures indicated that pathogen RNA makes up 0.2% or less of the total RNA in the infected kidney over the 48 hr time frame examined. These results show that this approach can reliably detect *C*. *albicans* RNAs of low abundance in invasive infection samples.

**Fig 1:**
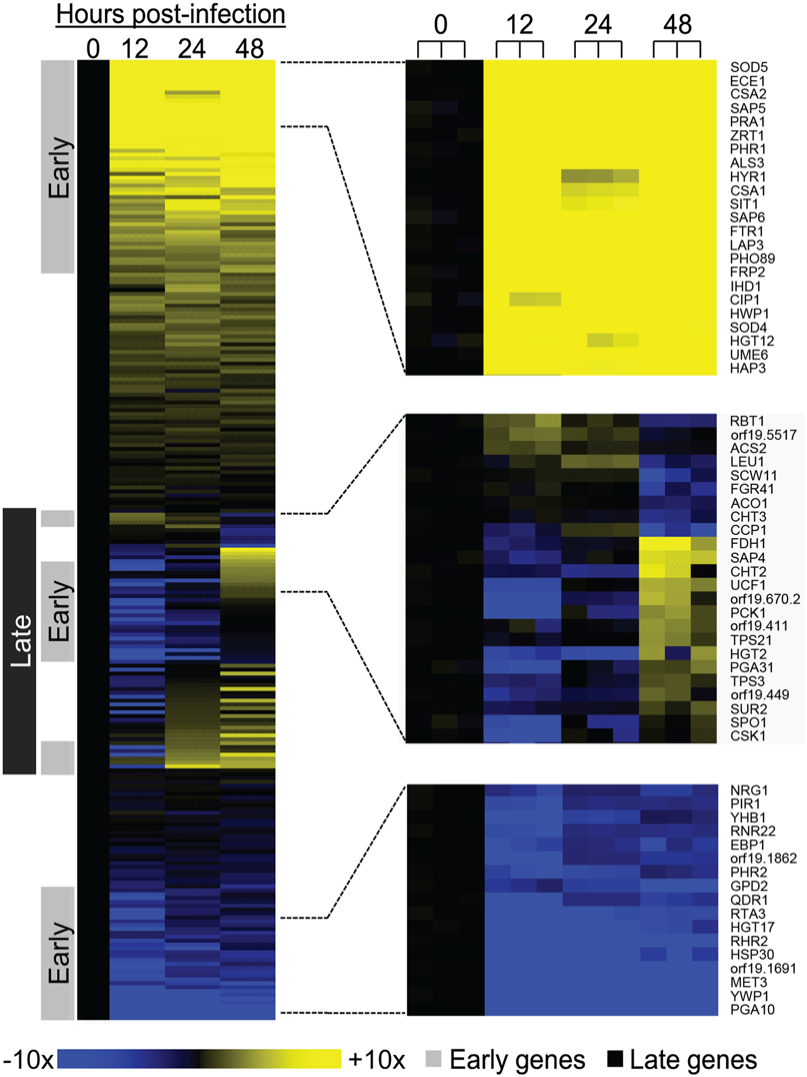
Expression of *C*. *albicans* environmentally responsive genes during invasive infection. Changes in expression levels during mouse kidney invasion for 248 *C*. *albicans* genes (S1 Data) are presented in a heat map format. Mean values of biological triplicates are shown for up-regulation (yellow) and down-regulation (blue) of genes at 12, 24, and 48 hr postinfection relative to mean inoculum levels (0 hr). Color saturation represents the extent of the expression change, with full saturation at 10-fold up- or down-regulation. (All heat maps in this article follow the same color scale.) Portions of the heat map are expanded to illustrate representative early up-regulated genes (top), late genes (middle), and early down-regulated genes (bottom). In these portions, individual samples are presented separately to illustrate reproducibility. We define early expression changes as significant differences between the inoculum and 12 hr sample. We define late expression changes as significant differences between the 12 and 48 hr samples. Significance refers to changes of ≥2-fold and a *p*-value < 0.05. The data for each sample were normalized to RNA levels from control gene *TDH3* before mean values were calculated. Our assignment criteria allow some dynamically regulated genes to fall into both the early and late expression classes. All numerical data for this figure are in S7 Data.

To assess changes in gene expression during infection, we compared the infected tissue samples to each other and to inoculum samples (stationary phase in yeast extract peptone dextrose medium [YPD]). The results revealed that *C*. *albicans* undergoes both early and late infection responses (Fig. 1; S1 Data). The early gene expression response comprises genes with RNA levels significantly different from the inoculum at 12 hr postinfection (*p* < 0.05 and ≥2-fold change in expression; Fig. 1). We found 65 up-regulated early genes and 74 down-regulated early genes. The late gene expression response comprises genes with RNA levels that are significantly different from the 12 hr time point at 48 hr postinfection (Fig. 1; S1 Data). We found 79 up-regulated late genes and 13 down-regulated late genes. These results indicate that *C*. *albicans* gene expression is dynamically regulated during invasive infection of a mammalian host.

**Fig 2:**
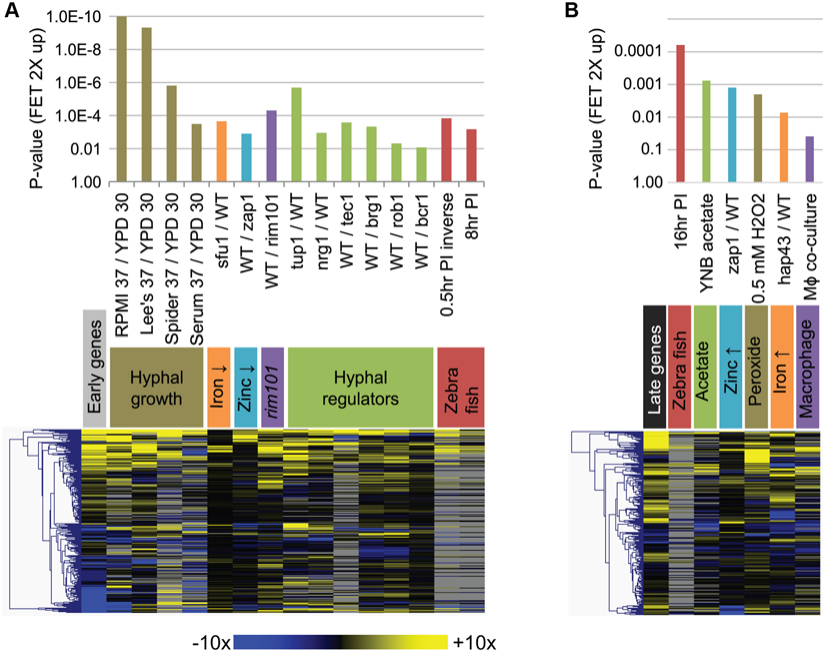
Comparison of gene expression profiles during invasive infection to other profiling datasets. Dataset comparisons were carried out with one sided Fisher's Exact Test (FET), using query gene sets from in vivo profiling data (applying a cutoff at 2-fold expression changes), and the entire set of nanoString probes (i.e., 248 environmental response genes) as a background set. Queries were matched to a database we assembled from 166 published expression datasets (S2 Data). (A). Representative matches to early up-regulated genes are shown with their respective *p*-values (top; higher bars indicate smaller *p*-values from FET, hence greater similarity between the two datasets), and in a heat map (bottom; same color scale as in Fig. 1; grey color indicates that no significant expression difference was reported and expression ratios were not readily available). (B). Representative matches to late up-regulated genes are shown with their respective *p*-values and in a heat map. Comprehensive FET results are available in S2 Data. Early and late gene sets are as defined in the Fig. 1 legend. All numerical data for this figure are in S7 Data.

To interpret the early and late gene expression changes, we compared our data to 166 published gene expression studies (S2 Data). We identified significant correlations among genes with expression changes of 2-fold or greater between datasets, as assessed with Fisher's exact test (FET). The most significant correlations were with genes up-regulated during invasive infection (Fig. 2A). Early up-regulated genes correlated well with genes expressed during the yeast-to-hyphal transition, including growth at 37° in Roswell Park Memorial Institute medium 1640 (RPMI), Lee's medium, Spider medium, or serum, all compared to growth at 30° in YPD. This correlation extended to the profiles of mutants defective in regulation of hyphal formation. For example, early up-regulated genes correlated with those up-regulated in a hyperfilamentous *tup1Δ/Δ* mutant (“tup1/wild type [WT]” dataset in Fig. 2A) and with those down-regulated in a nonfilamentous *tec1Δ/Δ* mutant (“WT/tec1” dataset in Fig. 2A). Early up-regulated genes also correlated well with genes that are repressed by iron acquisition gene repressor Sfu1 (“sfu1/WT” dataset) or activated by zinc acquisition gene activator Zap1/Csr1 (“WT/zap1” dataset). There was also a strong correlation with genes that depend upon transcription factor Rim101 for expression ("WT/rim101" dataset). Although Rim101-dependent genes overlap with yeast-to-hyphal genes, the correlation was significant with Rim101-dependent genes from an *nrg1Δ/Δ* background, in which hyphal gene expression changes were minimized [15]. There was also significant similarity to genes that were up-regulated mid-way through a zebrafish infection (“8 hr postinfection [PI]” dataset [16]), a point we return to in the Discussion. These correlations suggest the hypothesis that hyphal formation, iron limitation, zinc limitation, and Rim101 activation may be driving forces that govern early gene expression responses. We tested several of those inferences through the profiling of mutant strains presented below.

The late up-regulated genes correlated significantly with a distinct spectrum of gene expression responses (Fig. 2B). The late up-regulated genes correlated with genes up-regulated at late times during zebrafish infection. In addition, they correlated with genes induced by growth on acetate as carbon source, compared to glucose. The acetate response was assayed by Lorenz et al. [17] to model the metabolic state induced after phagocytosis by macrophages, and indeed the late infection response we describe here correlates with the response to co-culture with macrophages and to peroxide stress (Fig. 2B). Interestingly, we note that the zinc- and iron-limitation responses that were prominent early in infection begin to subside. This point is illustrated by the fact that early genes correlated with the "WT/zap1" dataset, while late genes correlate with its inverse, designated "zap1/WT." This reversal of iron and zinc responses may reflect a release of nutrients from damaged tissue, a phenomenon documented by Potrykus et al. with respect to iron levels [18]. These results suggest that the early and late infection environments are substantially different. The late gene expression responses may be driven by tissue damage and an inflammatory cell influx.

**Fig 3:**
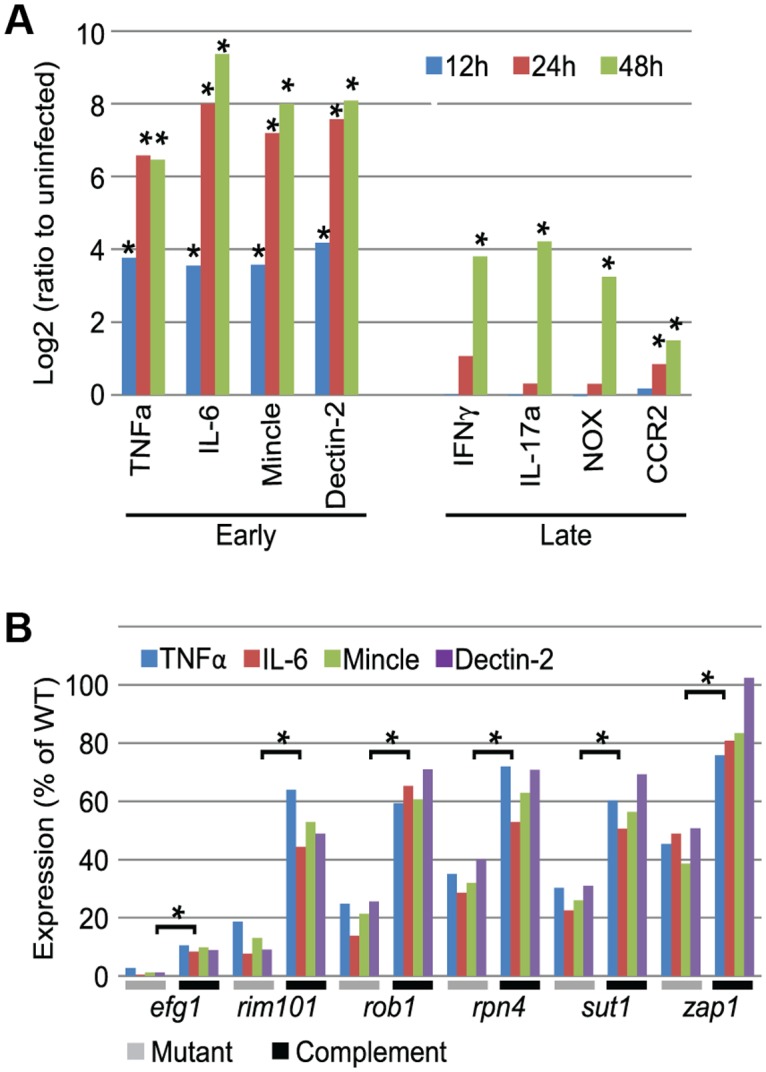
Mouse immune response gene expression during *Candida* infection. (A). RNA levels for 46 mouse immune response genes were assayed at 12, 24, and 48 hr postinfection in biological triplicates and were compared to uninfected kidney controls (S3 Data). Mouse genes that represent early and late expression classes are presented. We define early genes as those with significantly higher expression in 12 hr postinfection samples compared to uninfected samples. We define late genes as those with no significant difference in expression between uninfected and 12 hr postinfection samples, but with significantly higher expression in 48 hr postinfection samples compared to 24 hr postinfection samples. An asterisk indicates significant differences (*p*-value < 0.05) between successive time-course samples (uninfected samples versus 12 hr, 24 hr versus 12 hr, 48 hr versus 24 hr). (B). Mice were infected with the indicated transcription factor mutants and respective complemented strains; RNA levels for 46 mouse immune response genes were determined by nanoString at 24h postinfection (S3 Data). Induction of four early genes (the same genes shown in panel A for the wild-type strain) are shown here. All four genes were induced significantly more strongly by complemented strains than the respective mutants (*p*-value < 0.05), as indicated by the bracket and asterisk. *P*-values for expression of each gene are included in S3 Data. All numerical data for this figure are in S7 Data.

Two phases of host gene expression were also discernible (Fig. 3A; S3 Data). RNA levels for 46 mouse genes associated with fungal infection responses were assayed in each kidney sample. One set of genes was induced early in infection, reaching peak levels by 24 hr postinfection. This set included genes for pro-inflammatory cytokines (such as Tnfα and IL-6) and pattern recognition receptors (such as Mincle and Dectin-2), in keeping with the study by MacCallum at 24 hr postinfection [9]. These rapid responses are likely to be mediated by cells that are resident in the kidney. A second set of genes was induced late in infection, with significant expression increases between 24 and 48 hr (Fig. 3A). This second set included the neutrophil marker NOX (NADPH oxidase 1) and the inflammatory macrophage marker CCR2. The set also included genes whose expression is associated with T cells, such as interferon γ and IL-17a. These late responses probably reflect leukocyte migration into the kidney. Thus, both pathogen and host display distinct early and late temporal gene expression responses during invasive infection.

### Regulation of *C*. *albicans* Transcription during Infection

**Fig 4:**
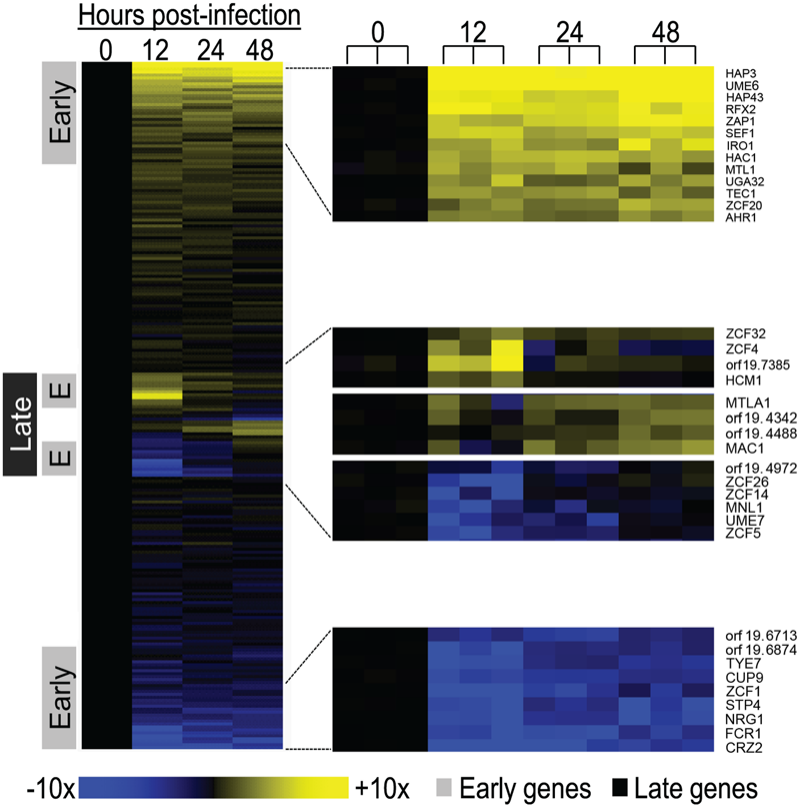
Expression of *C*. *albicans* transcription factor genes during invasive infection. Changes in expression levels during mouse kidney invasion for 231 *C*. *albicans* transcription factor genes (S4 Data) are presented in a heat map format. Mean values of biological triplicates are shown for up-regulation (yellow) and down-regulation (blue) of genes at 12, 24, and 48 hr postinfection relative to mean inoculum levels (0 hr). Portions of the heat map are expanded to illustrate representative early up-regulated genes (top), late genes (middle), and early down-regulated genes (bottom). In these portions, individual samples are presented separately to indicate reproducibility. The early and late gene classes were defined as described in the Fig. 1 legend. All numerical data for this figure are in S7 Data.

In order to identify regulators that govern gene expression during infection, we employed a probe set that included all 231 known or predicted *C*. *albicans* transcription factor genes. Early and late gene expression phases were evident among these genes (Fig. 4, S4 Data). Early up-regulated transcription factor genes included regulators of hyphal formation (Ume6, Tec1), zinc acquisition (Zap1), and iron acquisition (Hap43, Sef1). The late response included many transcription factors whose function has not been analyzed in detail previously. Functions of the early class of transcription factor genes that are known are consistent with the properties of environmentally responsive genes presented above.

Transcription factors that are expressed at high levels or significantly up-regulated during infection may govern pathogen proliferation and virulence. An analysis of previously studied transcription factors supports this idea (Table 1). Expression level was an excellent predictor of function. Specifically, among the 30 most highly expressed transcription factor genes at 48 hr postinfection, 18 have been found to be required for proliferation or virulence among 24 tested previously (75%). Among the 30 most weakly expressed transcription factor genes at this time point, one is required for proliferation or virulence among 12 tested previously (8%). This difference is highly significant (*p* < 0.001 by FET). The up- or down-regulation of expression was a weaker predictor of function. Among the 30 most highly up-regulated transcription factor genes (comparing 48 hr postinfection to the inoculum samples), 15 are required for proliferation or virulence among 20 tested previously (75%). Among the 30 most down-regulated transcription factor genes, seven are required for proliferation or virulence among 16 tested previously (44%). There is no significant difference between these expression classes (*p* > 0.08 by FET). Therefore, the transcription factor gene expression level, rather than its regulatory response, is the most discerning predictor of function in this infection model. However, transcription factors that are most highly up-regulated or most highly expressed are equally likely to have clear roles in proliferation and virulence (*p* = 1.0 by FET).

**Table 1:** Transcription factor gene expression and function in vivo.

Gene class^1^		Virulence/Function^2^
Highly expressed	AHR1	reduced/f,s
	BRG1	reduced/f,s
	CAP1	reduced/s
	EFG1	reduced/f,s
	HAP3	reduced
	HAP43	reduced/s
	IRO1	reduced/f
	NDT80	reduced/f,s
	RFG1	reduced/f,s
	RFX2	reduced/f,s
	RIM101	reduced/f,s
	RPN4	reduced/s
	SEF1	reduced/f
	TEC1	reduced/f,s
	TUP1	reduced/f,s
	UME6	reduced/f,s
	ZAP1	reduced/f,s
	orf19.5848	reduced
	HAC1	n.a./f,s
	MBF1	n.a.
	MDM34	n.a.
	MET28	n.a.
	STP2	n.a./f,s
	orf19.4488	n.a.
	CPH2	normal/f,s
	GAL4	normal
	GCN4	normal/f,s
	GIS2	normal
	TYE7	normal/f,s
	ZCF39	normal/f
Weakly expressed	EFH1	reduced/f
	CRZ2	n.a./s
	MNL1	n.a./s
	MTLA2	n.a.
	REP1	n.a.
	RME1	n.a.
	TRY6	n.a.
	WOR1	n.a./f
	ZCF13	n.a./f,s
	ZCF19	n.a.
	ZCF22	n.a.
	ZCF4	n.a.
	ZCF5	n.a./f
	orf19.7385	n.a.
	orf19.513	n.a.
	orf19.3407	n.a./s
	orf19.259	n.a.
	orf19.226	n.a.
	orf19.1178	n.a.
	FGR17	normal/f,s
	SFU1	normal/s
	TEA1	normal/f
	UME7	normal/f
	WOR2	normal/f
	ZCF14	normal/f
	ZCF26	normal
	orf19.4972	normal
	orf19.4195	normal
	orf19.217	normal
	orf19.1577	normal
Up-regulated	AHR1	reduced/f,s
	BCR1	reduced/f,s
	CPH1	reduced/f,s
	HAP3	reduced
	HAP43	reduced/s
	IRO1	reduced/f
	RFG1	reduced/f,s
	RFX2	reduced/f,s
	RIM101	reduced/f,s
	SEF1	reduced/f
	SUT1	reduced
	TEC1	reduced/f,s
	UME6	reduced/f,s
	ZAP1	reduced/f,s
	orf19.5848	reduced
	HAC1	n.a./f,s
	HAP2	n.a.
	INO4	n.a.
	MAC1	n.a./f
	MTLA1	n.a.
	MTLα1	n.a.
	SEF2	n.a.
	ZCF20	n.a./s
	ZCF31	n.a.
	orf19.4488	n.a.
	UGA32	normal/f
	ZCF38	normal
	ZNC1	normal
	orf19.3928	normal
	orf19.217	normal
Down-regulated	ASH1	reduced/f,s
	EFG1	reduced/f,s
	EFH1	reduced/f
	GZF3	reduced/f,s
	LYS144	reduced
	NDT80	reduced/f,s
	NRG1	reduced/f,s
	CIRT4B	n.a.
	CRZ2	n.a./s
	GRF10	n.a./f
	HAL9	n.a./f
	RAP1	n.a./f,s
	RME1	n.a.
	STP4	n.a./f
	UGA3	n.a.
	UPC2	n.a./s
	ZCF13	n.a./f,s
	ZFU2	n.a./f
	ZPR1	n.a./s
	orf19.6713	n.a.
	orf19.6227	n.a.
	CUP9	normal/f
	FCR1	normal/f,s
	GIS2	normal/s
	SFU1	normal/s
	TYE7	normal/f,s
	WOR2	normal/f
	ZCF1	normal
	orf19.6874	normal/f
	orf19.5026	normal

^1^ The top 30 transcription factor genes in each indicated category are based on expression data at 48 hr postinfection (S4 Data). "Highly expressed" or "Weakly expressed" refers to the rank order of nanoString counts at 48 hr postinfection. "Up-regulated" or "Down-regulated" refers to the fold change in nanoString counts at 48 hr postinfection compared to inoculum samples.

^2^ Virulence or proliferation of a null mutant in the respective gene was compiled from information at the Candida Genome Database; n.a. = data not available. Data for *RPN4* and *SUT1* are from this study. Function was summarized from association with Gene Ontology (GO) terms "filamentous growth" (f) or "response to stress" (s).

### Profile of the Attenuated *rim101Δ/Δ* Transcription Factor Mutant

**Fig 5:**
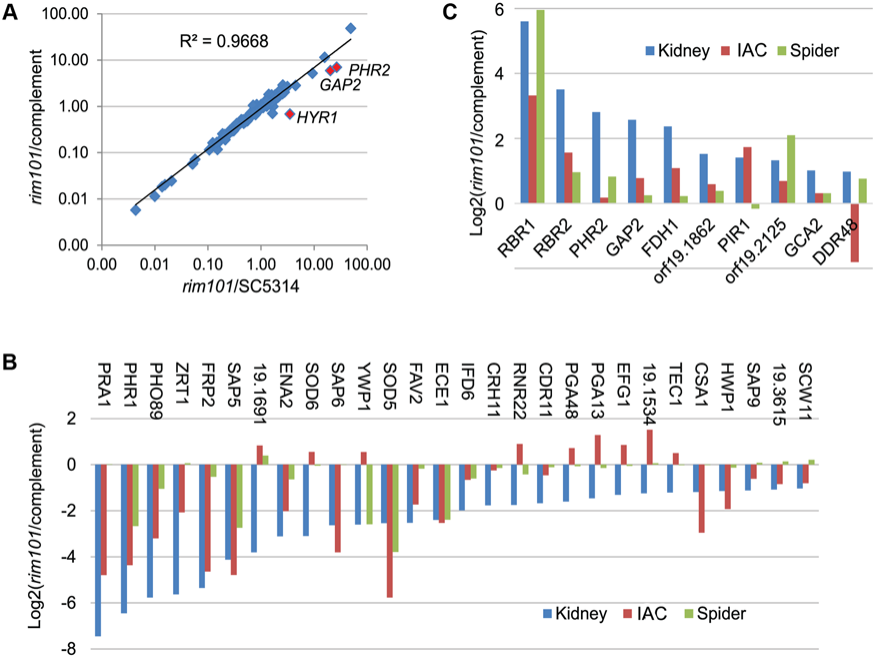
Rim101-dependent gene regulation during invasive infection. RNA levels for 148 *C*. *albicans* environmental response genes were determined by nanoString at 24 hr postinfection for the *rim101* mutant and complemented strains (S5 Data). (A). Expression ratios are plotted for each gene in *rim101Δ/Δ* versus wild type (X axis) and *rim101Δ/Δ* versus complemented strain (Y axis). Three genes (red data points) have significantly different expression ratios in the two comparisons. (B). Expression ratios are presented for all genes significantly down-regulated in the *rim101Δ/Δ* strain relative to the complemented strain during kidney infection (blue bars; ≥2-fold change and *p*-value < 0.05). The expression ratios for the same genes in the same strains during intra-abdominal candidiasis (IAC) infection (red bars; reported in [12]) or during in vitro growth in Spider medium (green bars) are displayed. Complete data are in S5 Data. (C). Expression ratios are presented for all genes significantly up-regulated in the *rim101Δ/Δ* strain relative to the complemented strain during kidney infection (blue bars; ≥2-fold change and *p*-value < 0.05), during abdominal infection (red bars; reported in [12]) or during in vitro growth in Spider medium (green bars) are displayed. Complete data are in S5 Data. All numerical data for this figure are in S7 Data.

We sought to understand regulatory relationships during infection, and we began this analysis with the transcription factor Rim101. We chose *RIM101* because it is the most highly expressed transcription factor gene at 48 hr postinfection; its RNA levels comprise over 3% of the total RNA levels for all 231 transcription factor genes (S4 Data). Rim101 clearly functions during infection: early up-regulated genes correlate with known Rim101-dependent genes, and prior studies show that it is required for virulence in the mouse disseminated infection model, where it promotes both hyphal formation and proliferation [19]. In order to define the gene expression basis for the *rim101Δ/Δ* mutant virulence defect, we assayed expression of 148 environmentally responsive genes by the *rim101Δ/Δ* mutant and *rim101Δ/Δ+pRIM101* complemented strain at 24 hr postinfection (S5 Data). The *rim101Δ/Δ* mutant presented significantly reduced accumulation of 33 RNAs (≥2-fold change and *p* < 0.05, including *RIM101* itself), and increased expression of 14 RNAs, compared to the wild-type strain (S5 Data). RNA accumulation in the complemented strain closely mirrored that of the wild type (Fig. 5A). Specifically, RNA accumulation levels were restored by the complementing *RIM101* allele for all genes except *GAP2*, *PHR1*, and *HYR1* (*p* < 0.05). In the complemented strain, expression of *GAP2* and *PHR1* trended toward the wild-type level, thus suggesting that these few gene expression differences from the wild type are the result of reduced gene dosage of *RIM101* alleles in the complemented strain compared to the wild type, as documented previously for these strains [19].

Genes that were down-regulated in the *rim101Δ/Δ* mutant included many that are induced during hyphal growth (such as *HWP1*, *ECE1*, *SOD5*, and *SAP5*). Their reduced expression in the mutant is consistent with its deficiency in hyphal growth in vivo [19]. Two transcription factor genes that are required for hyphal growth, *EFG1* and *TEC1*, were also down-regulated in the mutant and may account for its failure to form hyphae and express hyphal genes. We note that the few direct Rim101 target genes assayed have altered RNA levels in the mutant, including Rim101-activated genes *PHR1* and *PRA1*, as well as Rim101-repressed gene *PHR2* [20,21]. Overall, these in vivo profiling data are consistent with known features of the *rim101Δ/Δ* pathogenicity defect and the molecular data about the Rim101 protein.

The gene expression impact of the *rim101Δ/Δ* mutation has been characterized extensively in vitro, and we noted several differences between published in vitro profiles and our in vivo data. Such differences can arise from the assay platform, and we thus examined the *rim101Δ/Δ* mutant and *rim101Δ/Δ+pRIM101* complemented strain under hypha-inducing conditions in vitro (Spider medium at 37°C) with the nanoString platform. We examined expression of 144 environmentally responsive genes (S5 Data). In this set, 37 genes responded significantly (≥2-fold change, *p* < 0.05) to Rim101 in vivo, 11 genes responded significantly to Rim101 in vitro, and only eight responded significantly to Rim101 under both conditions (compare blue and green bars in Fig. 5B and Fig. 5C). The genes that responded significantly to Rim101 only in vivo include several with roles in pathogenicity (*EFG1*, *HWP1*, *SAP6*, and *TEC1*). These results emphasize that the invasive infection environment can alter the spectrum of genes that respond to a transcription factor, and they suggest that such alterations have the potential to influence virulence.

We have recently analyzed the *rim101Δ/Δ* mutant and *rim101Δ/Δ+pRIM101* complemented strain on the nanoString platform during abdominal infection [12]. Among 144 genes assayed in both infection models, we found 46 genes that responded significantly to Rim101 during abdominal infection, and only 15 responded similarly in both infection models (Fig. 5B and Fig. 5C, blue and red bars). Notably, the hyphal regulatory genes *EFG1* and *TEC1* did not require Rim101 for expression during abdominal infection. Therefore, the site of infection can affect the relationship between a transcription factor and its target genes.

### Profile of the Attenuated *efg1Δ/Δ* Transcription Factor Mutant

We also examined the gene expression impact of a defect in the transcription factor Efg1. An *efg1Δ/Δ* mutant was among the first attenuated *C*. *albicans* strains characterized [22,23]. Efg1 governs numerous pathogenicity-related phenotypes, including adherence to diverse cells and substrates, formation of hyphae at 37°C, colonization of the gastrointestinal tract, and antifungal drug susceptibility [14,24]. The impact of *efg1Δ/Δ* mutations has been assessed on the *C*. *albicans* transcriptome during growth in vitro [25], but never in vivo.

**Fig 6:**
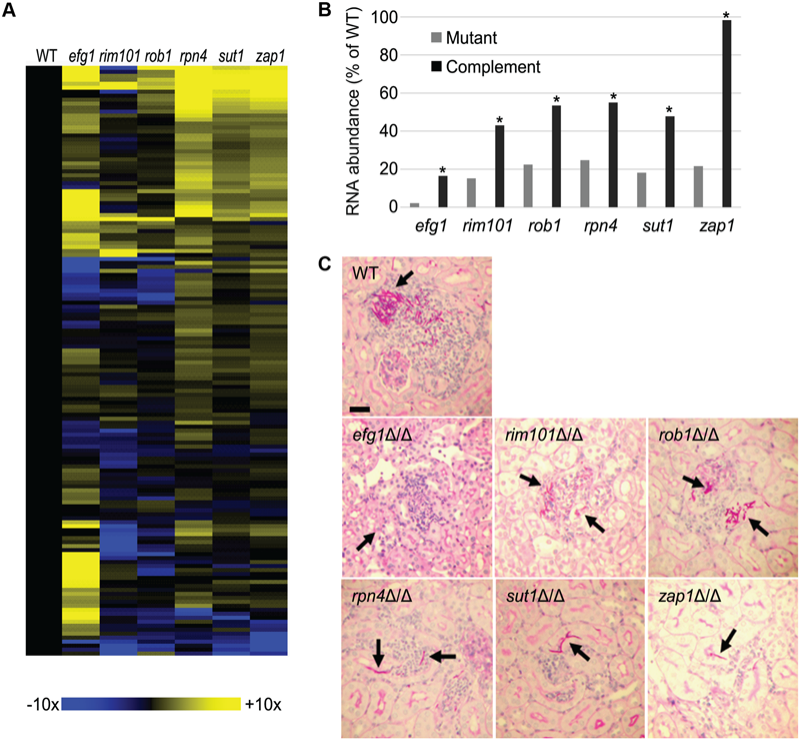
Comparison of *C*. *albicans* proliferation-defective transcription factor mutants. (A). Heat map representation of gene expression ratios for 148 environmentally responsive genes at 24 hr postinfection (*rim101Δ/Δ*, *rob1Δ/Δ*, *rpn4Δ/Δ*, *sut1Δ/Δ*, or *zap1Δ/Δ*, each relative to the wild type) or 48 hr postinfection (*efg1Δ/Δ*, relative to the wild type). Complete data are in S5 Data. (B). Yield of *C*. *albicans* RNA relative to total kidney RNA for each mutant and complemented strain at 24 hr postinfection. Asterisks mark significant differences (*p*-value < 0.05) between mutants and respective complemented strains. (C). Histopathology images of kidney sections. All samples were at 24 hr postinfection. Arrows indicate fungal cells. The scale bar corresponds to 50 microns. For panel A, the specific strains used and the dose of viable cells per mouse, as determined by plating the inocula, were: CW 696 (wild type) 8.4 × 10^5^; CW730 (*rob1*) 10.2 × 10^5^; DAY25 (*rim101*) 11.1 × 10^5^; CW1018 (*efg1*) 9 × 10^5^; CW792 (*rpn4*) 7.9 × 10^5^; CW756 (*zap1*) 8.8 × 10^5^; CW704 (*sut1*) 8.6 × 10^5^. All numerical data for this figure are in S7 Data.

We confirmed that the proliferation of an *efg1Δ/Δ* mutant was severely defective in the mouse disseminated infection model, yielding overall RNA levels too low to detect expression of many *C*. *albicans* genes at 24 hr postinfection (Fig. 6B) and a greatly diminished host response (Fig. 3B). Therefore, we assayed *C*. *albicans* gene expression in an *efg1Δ/Δ* mutant and *efg1Δ/Δ+pEFG1* complemented strain at 48 hr postinfection. Among 148 environmentally responsive genes assayed, we found 26 genes that were significantly down-regulated (including *EFG1* itself) and 63 genes that were significantly up-regulated (≥2-fold and *p* < 0.05), compared to the wild-type strain (Fig. 6A; S5 Data). Presence of one *EFG1* allele in the complemented strain increased *EFG1* expression to 26% of the wild-type level, and permitted partial or complete restoration of environmentally responsive gene expression levels (S3 Fig.; S5 Data). This dosage effect is consistent with prior studies that document haploinsufficiency for *EFG1* [26]. The 26 down-regulated genes included many hyphal genes (for example, *ALS3*, *ECE1*, *HWP1*, *IHD1*, and *SOD5*), as expected from profiling of an in vitro–grown *efg1Δ/Δ* mutant [25]. Among the 63 up-regulated genes, only four were up-regulated in the in vitro *efg1Δ/Δ* characterization [25], and their fold-change was much greater in vivo than in vitro. These results indicate that the *efg1Δ/Δ* mutant is defective in expression of hyphal genes during proliferation in vivo, as expected from its previous phenotypic characterization. In addition, the *efg1Δ/Δ* mutant, like the *rim101Δ/Δ* mutant, has impact on gene expression during proliferation in vivo that is distinct from what is observed in vitro.

### Functional Relationships among Virulence Regulators

In order to visualize gene expression alterations associated with virulence defects in a broader context, we profiled a panel of mutants that are defective in proliferation in vivo. We selected a *zap1Δ/Δ* mutant because many known Zap1-dependent genes are up-regulated during infection, as discussed above. The *zap1Δ/Δ* mutant has been shown to be defective in proliferation previously [27], a phenotype that was confirmed by its reduced RNA levels in infected tissue (Fig. 6B) and a diminished host response (Fig. 3B). We also made mutations in three highly expressed or up-regulated transcription factor genes that had not been studied previously in a disseminated infection model: *ROB1* (orf19.4998), *RPN4* (orf19.1069), and *SUT1* (orf19.4342). Each mutation caused reduced proliferation in vivo, as indicated by a decreased fungal RNA yield (Fig. 6B) and host response (Fig. 3B). Histopathology examination of kidney sections at 24 hr postinfection showed that the attenuated mutants were able to invade the kidney parenchyma, though fungal cells were much less abundant than observed in mice infected with the wild-type strain (Fig. 6C). Complementation with a wild-type copy of each gene led to increased fungal RNA yield and host response gene expression (Fig. 6B and Fig. 3B). Therefore, defects in *ROB1*, *RPN4*, and *SUT1* cause defects in proliferation in vivo.

In order to identify the virulence pathways that are governed by these transcription factors, we assayed expression of 148 *C*. *albicans* environmentally responsive genes at 24 hr postinfection in the wild type and the attenuated transcription factor mutant strains (Fig. 6A; S5 Data), and compared the results to the *rim101Δ/Δ* and *efg1Δ/Δ* data presented above. Gene expression alterations in each mutant were largely restored in the respective complemented strains (S3 Fig.; S5 Data), thus indicating that the mutation introduced into each strain was the cause of the gene expression alteration. Only three genes had significantly altered expression (≥2-fold, *p* < 0.05) in all mutants, the up-regulated cell wall or secreted protein genes *GCA2*, *RBR1*, and *RBR2*. Although no firm conclusion can be drawn from so few genes, the results suggest that attenuated mutants may undergo a common cell surface alteration. Prior and current studies indicate that Rim101, Rob1, and Efg1 are required for hyphal formation, so we looked for common gene expression alterations in those three strains. Common down-regulated genes included *CRH11*, *ECE1*, *FAV2*, *HWP1*, *IFD6*, *SAP5*, and *SAP6*, most of which are associated with hyphal morphogenesis. Therefore, this feature of the in vivo gene expression profile is consistent with prior in vitro analysis of the hyphal morphogenesis program and the mutant phenotypes observed in vivo.

Three of the mutants, *rpn4Δ/Δ*, *sut1Δ/Δ*, and *zap1Δ/Δ*, had extremely similar gene expression profiles (Fig. 6A top right; S5 Data). These results were unexpected because these three *C*. *albicans* mutants have distinct phenotypes in vitro. For example, on low-zinc medium, the *zap1Δ/Δ* mutant fails to grow, while the *rpn4Δ/Δ* and *sut1Δ/Δ* mutants grow well (S4 Fig.). Also, during growth in vitro, the gene expression alterations of the three mutants have little similarity (S4 Fig.). We considered two explanations for our observations. One possibility is that Rpn4, Sut1, and Zap1 act in a pathway that operates in the invasive infection environment to govern proliferation; in vitro growth conditions may alter their spectra of target genes. A second possibility is that Rpn4, Sut1, and Zap1 are functionally unrelated, and the in vivo gene expression profiles of the three mutants represent general proliferation-defective responses.

**Fig 7:**
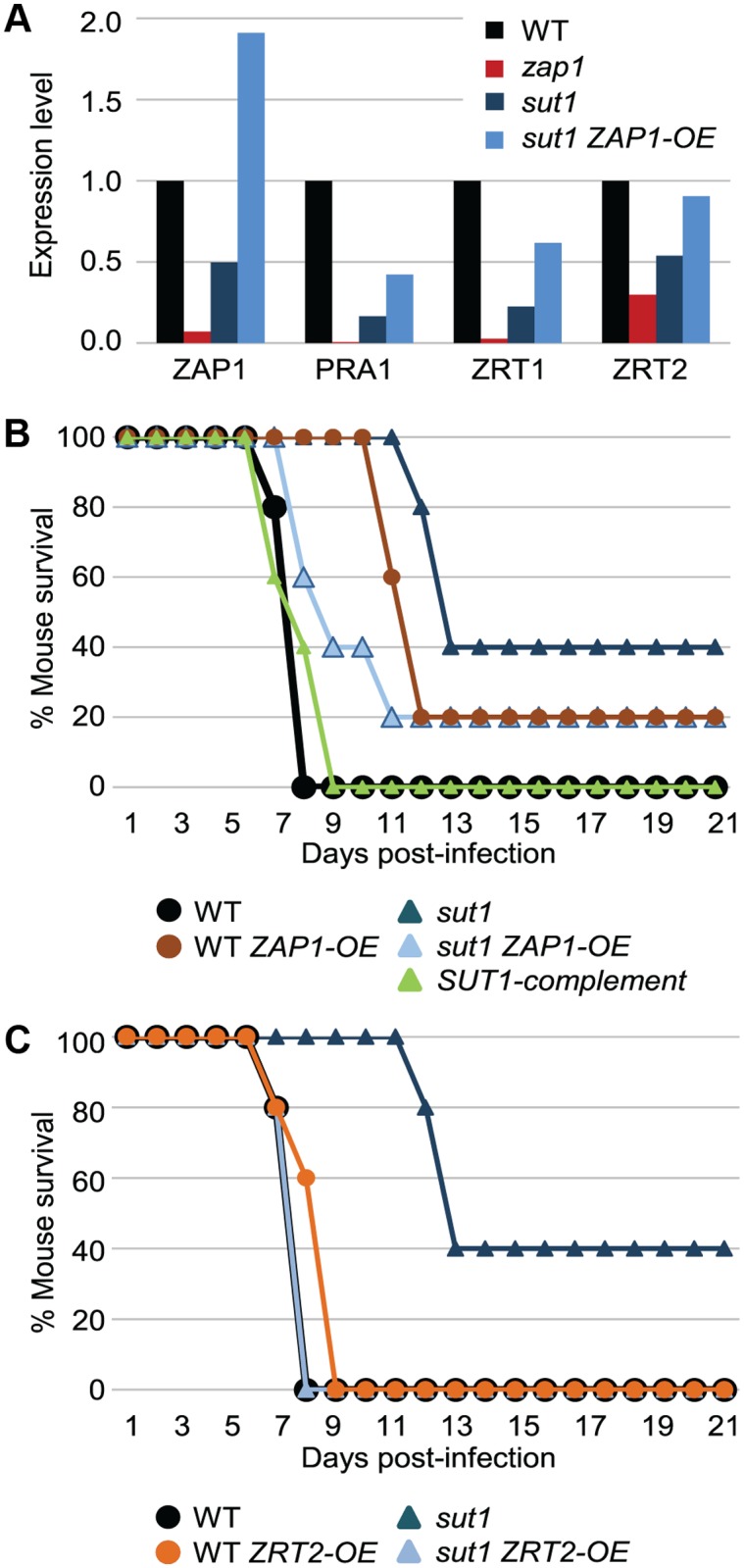
Effect of *ZAP1* and *ZRT2* overexpression in a *sut1Δ/Δ* mutant. (A). Expression of zinc acquisition genes *ZAP1*, *PRA1*, *ZRT1*, and *ZRT2* was measured by nanoString at 24 hr postinfection in the wild type, the *zap1Δ/Δ* mutant, the *sut1Δ/Δ* mutant, and the *sut1Δ/Δ* mutant that overexpresses *ZAP1*. The mean of triplicate determinations is shown. (B). Mouse survival was determined after inoculation with the wild-type strain, the *sut1Δ/Δ* mutant, the *sut1Δ/Δ+pSUT1* complemented strain, the wild-type strain that overexpresses *ZAP1*, and the *sut1Δ/Δ* mutant that overexpresses *ZAP1*. Mouse survival was significantly better after infection with the *sut1Δ/Δ* mutant than after infection with the wild-type strain, the *sut1Δ/Δ+pSUT1* complemented strain, or the *sut1Δ/Δ* mutant that overexpresses *ZAP1* (*p* < 0.05 by the log-rank test). (C). Mouse survival was determined after inoculation with the wild-type strain that overexpresses *ZRT2* and the *sut1Δ/Δ* mutant that overexpresses *ZRT2*. Survival data after inoculation with the wild-type strain and the *sut1Δ/Δ* mutant from panel B are also included for comparison; all infections shown in panels B and C were carried out in parallel. The specific strains used and the dose of viable cells per mouse, as determined by plating the inocula, were: CW696 (wild type) 4.4 × 10^5^; CW704 (*sut1*) 4.9 × 10^5^; CW1035 (*SUT1* complement) 3.2 × 10^5^; WX134 (WT *ZAP1-OE*) 3.35 × 10^5^; WX102 (*sut1 ZAP1-OE*) 3.65 × 10^5^; WX144 (*sut1 ZRT2-OE*) 4.85 × 10^5^; WX137 (WT *ZRT2-OE*) 3.75 × 10^5^. Complete genotypes are given in S1 Table. All numerical data for this figure are in S7 Data.

We focused on Sut1 and Zap1 to determine whether there may be a functional relationship in vivo. We chose those two transcription factors because, during proliferation in vivo, the *sut1Δ/Δ* mutant had reduced RNA levels for *ZAP1* and the Zap1-dependent genes *PRA1*, *ZRT1*, and *ZRT2* (Fig. 7A). Therefore, we considered the specific hypothesis that the *sut1Δ/Δ* phenotypic defect during infection arises from its *ZAP1* expression defect. This model predicts that overexpression of *ZAP1* will suppress the defects of a *sut1Δ/Δ* mutant. We created a *ZAP1* overexpression allele by fusing the *ZAP1* coding region to a strong *TDH3* promoter in a *sut1Δ/Δ* strain. The *sut1Δ/Δ TDH3-ZAP1* strain expressed zinc acquisition genes *PRA1*, *ZRT1*, and *ZRT2* during infection at higher levels than the *sut1Δ/Δ* mutant (Fig. 7A). In order to assess the biological significance of the Sut1-Zap1 relationship, we made use of the finding that the *sut1Δ/Δ* mutant is defective in virulence (Fig. 7B). Specifically, mice infected with the *sut1Δ/Δ* mutant survived substantially longer than those infected with the wild type or complemented strain. The *sut1Δ/Δ TDH3-ZAP1* strain displayed much greater virulence than the *sut1Δ/Δ* mutant, causing host lethality with nearly wild-type kinetics (Fig. 7B). We considered the possibility that *ZAP1* overexpression may cause a nonspecific increase in virulence. However, an otherwise wild-type strain that carried the *TDH3-ZAP1* allele had reduced virulence rather than increased virulence (Fig. 7B). These findings are consistent with the model that Sut1 is necessary for pathogenicity because it is required for *ZAP1* expression during infection. To test the specific hypothesis that the *sut1Δ/Δ* mutant is attenuated because it is defective in zinc uptake, we assayed virulence of strains that express the zinc transporter gene *ZRT2* from the *TDH3* promoter. The *TDH3-ZRT2* allele did not alter virulence in a wild-type background, but fully restored virulence of a *sut1Δ/Δ* mutant (Fig. 7C). This finding proves that the zinc transporter expression defect of the *sut1Δ/Δ* mutant is the cause of its virulence defect. Therefore, Sut1 is required for zinc acquisition in the invasive infection environment.

### In Vivo Response to Antifungal Therapy

**Fig 8:**
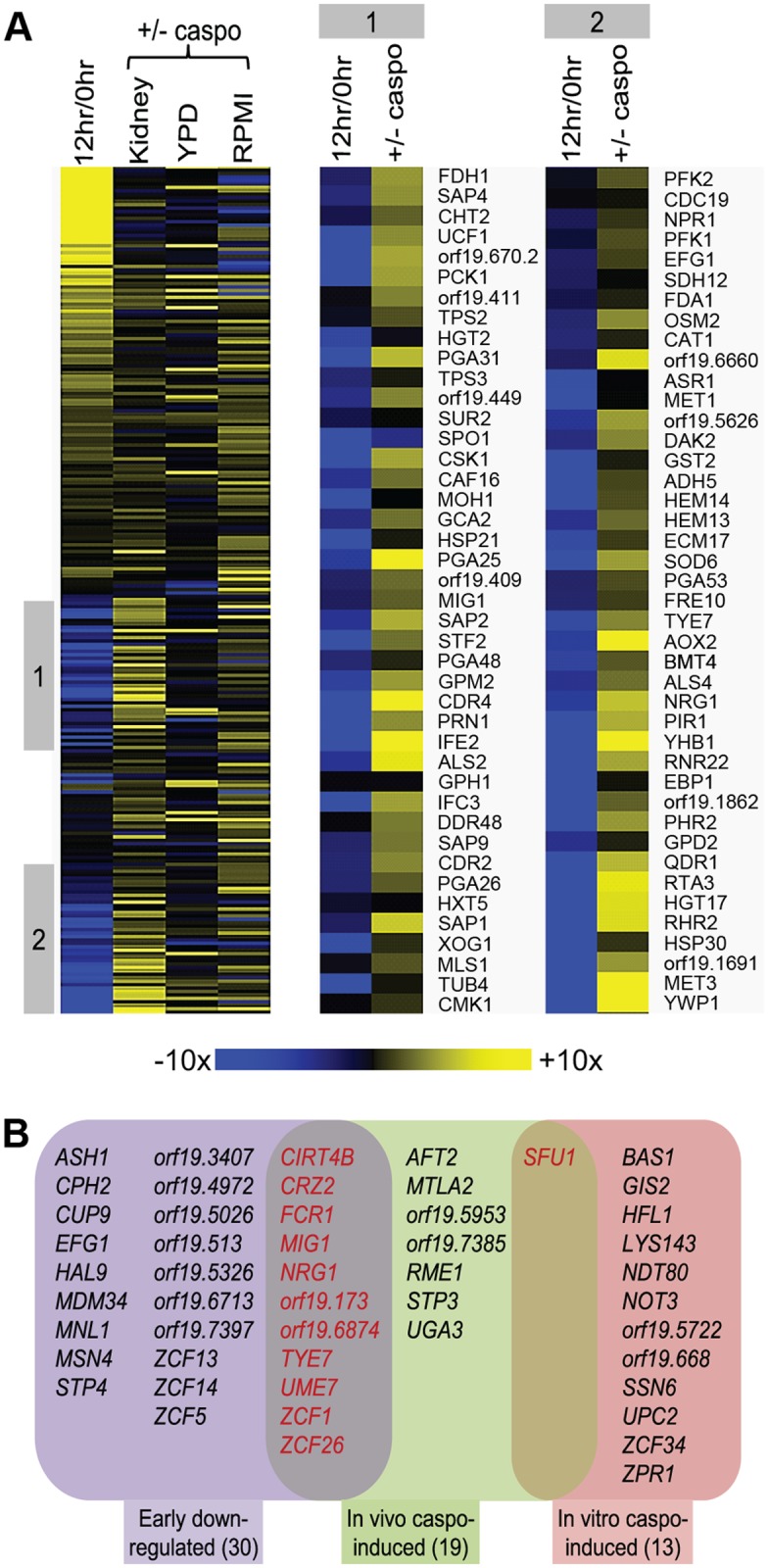
Gene expression response to caspofungin treatment during infection. (A). Changes in expression levels for 248 *C*. *albicans* environmentally responsive genes are presented for caspofungin treated versus untreated cells at 24 hr postinfection (“Kidney,” S6 Data), in vitro in YPD at 30°C (“YPD,” from [30]), and in vitro in RPMI at 37°C (“RPMI,” S6 Data). These environmentally responsive genes are the same ones for which expression was measured during the time-course of infection depicted in Fig. 1. For comparison, the expression ratios of the same genes at 12 hr postinfection relative to the inoculum are shown (“12 hr/0 hr,” from Fig. 1). The data are presented in heat map format. Regions “1” and “2” are expanded on the right to make gene names legible. (B). Expression levels for 231 *C*. *albicans* transcription factor genes were measured for caspofungin treated versus untreated cells at 24 hr postinfection (“In vivo caspo-induced,” S6 Data) and in vitro in RPMI at 37°C (“In vitro caspo-induced,” S6 Data). Significantly up-regulated transcription factor genes are listed (≥2-fold change and *p* < 0.05). For comparison, the significantly down-regulated transcription factor genes at 12 hr postinfection are listed (“Early down-regulated,” S6 Data). All numerical data for this figure are in S7 Data.

Caspofungin, a cell wall inhibitor, is an extremely effective antifungal drug [6]. The gene expression response to caspofungin treatment has been assayed during growth in vitro [28–30], but not under infection conditions. We reasoned that the response to caspofungin may be different in vivo. Therefore, we assayed expression of the 248 environmentally responsive genes 2 hr after caspofungin administration in mice that had already been infected for 24 hr. There was no detectable decline in fungal cell number after this brief treatment time, but an extensive gene expression response was manifested (S6 Data). A much broader set of genes was induced by caspofungin in vivo than had been detected through previous in vitro studies (Fig. 8A). Specifically, 44 of the genes assayed showed significantly increased RNA accumulation (≥2-fold, *p* < 0.05) after caspofungin treatment in vivo compared to untreated infection samples. Several induced genes specify cell wall or secreted proteins (*ALS4*, *ALS9*, *GCA2*, *PGA13*, *PGA26*, *PGA31*, *PGA37*, *PHR2*, *PIR1*, *RBR2*, *SAP1*, and *SAP9*), and others specify enzymes that function in glucose generation (*PCK1*, *GPM2*, and *DAK2*). Thus, the in vivo response suggests that the cell wall is restructured in response to the drug through an alteration of cell wall protein composition and an increase in β-glucan synthesis.

The gene expression response to caspofungin during infection showed little similarity to the previously characterized response during growth in vitro (Fig. 8A). Among significantly regulated genes from one previous in vitro study [30], 32 could be detected by our nanoString probes, and only four were regulated in parallel (up-regulated: *PHR2*, *RTA4*, *SAP9*; down-regulated: *FGR41*). Previous studies had been carried out with yeast-form cells and with a microarray platform, both of which may contribute to the divergence of results. Therefore, we assayed the gene expression response to caspofungin during hyphal growth conditions (RPMI, 37°C) with the same 248 nanoString probes used for in vivo profiling. We found a broad response to caspofungin under these in vitro conditions, with 19 down-regulated genes and 32 up-regulated genes (Fig. 8A; S6 Data). The up-regulated genes included numerous cell wall or secreted protein genes (*ALS1*, *CHT2*, *CRH11*, *DFG5*, *ECM331*, *KRE1*, *PGA17*, *PGA26*, *PHR1*, *RBR1*, and *SOD4*) as well as glucose generation genes (*FBP1*, *ICL1*, and *PCK1*). Surprisingly, though, there was still limited similarity to the in vivo response to caspofungin: seven genes were regulated in parallel (up-regulated: *PGA26*, *PCK1*, *MET3*, and *YHB1*; down-regulated: *ATO2*, *CIP1*, and *LAP3*). These results indicate that the infection environment has considerable impact on the gene expression response to caspofungin antifungal treatment.

In order to determine whether any previously characterized gene expression responses resemble the in vivo caspofungin response, we compared in vivo caspofungin-responsive genes to our database of gene expression data. We found striking overlap between caspofungin up-regulated genes and those genes that are repressed at 12 hr postinfection (Fig. 8A; *p* < 0.0001 by FET). Specifically, among 44 genes that were up-regulated in vivo in response to caspofungin, 35 genes had been repressed within 12 hr postinfection (Fig. 8A). These 35 genes include several with roles in cell wall biogenesis and integrity, such as *PGA31*, *PHR2*, *PIR1*, and *SAP9*. Down-regulation of these genes at an early time during infection may render infecting cells more vulnerable to cell wall inhibitors than in vitro-grown cells.

The gene expression responses to caspofungin may differ in vivo and in vitro because of a difference in the transcription factors that mediate the response under each condition. Therefore, we examined the transcription factor genes that are induced by caspofungin under the two conditions (Fig. 8B; S6 Data). Under infection conditions, we detected induction of 18 transcription factor genes (≥2-fold induction, *p* < 0.05). Under in vitro conditions (RPMI, 37°C), we detected induction of 13 transcription factor genes. The two sets of induced genes had minimal overlap; only iron regulator *SFU1* was induced under both conditions. However, the caspofungin up-regulated transcription factor genes included 11 genes that were down-regulated within 12 hr postinfection. It seems reasonable that the different gene expression responses to caspofungin treatment in vivo and in vitro reflect the difference in transcription factor gene responses that we have discovered here.

## Discussion

Our knowledge of many pathogens comes primarily from in vitro analyses, though their behavior during interaction with the host is what we hope to understand. Here we have captured a slice of pathogen gene expression during invasive infection, thus providing a window into pathogen behavior in the infection environment. Our results show that there are two clear phases of infection, and that the gene expression features of each phase agree well with current thinking about the infection environment. However, we find that regulatory relationships defined through in vitro experiments are modified in the environment of invasive infection, and that those modifications can have functional impact. In addition, we have identified an unanticipated feature of drug-responsive gene expression that may be a useful consideration in future therapeutic development.

### Infection Portrait from Gene Expression Dynamics

The expression profile reported here is, to our knowledge, the largest time-course analysis of *C*. *albicans* infection of a mammalian host. The results indicate that there are both early and late gene expression changes during infection. Early genes underscore the importance of many driving forces in infection that have been deduced from previous studies of *C*. *albicans* and other invasive pathogens: hyphal formation and limitation for iron and zinc [31,32]. Late genes reflect responses that have been logically inferred from interactions with innate immune cells: oxidative stress and the consequences of phagocytosis by macrophages [32,33]. In addition, late responses suggest that iron and zinc limitation become less severe as infection proceeds, as reported for iron availability by Potrykus et al. [18]. Therefore, the overall features of gene expression during infection that we describe here fit well with the current understanding of *C*. *albicans* infection biology.

The changes in carbon metabolic gene expression we observe during infection suggest that invasive cells shift from glycolysis to gluconeogenesis. Early up-regulated genes included four genes that specify subunits of pyruvate dehydrogenase, which is needed for carbon flux from glycolysis into the tricarboxylic acid cycle. Early down-regulated genes included the glycerol biosynthetic genes *GPD2* and *RHR2*, a likely reflection of metabolic feedback from high levels of the kidney osmoprotectant glycero-phosphocholine [34], which is utilized by *C*. *albicans* during infection [35]. Also down-regulated early was the *PCK1* gene, which specifies the gluconeogenic enzyme phosphoenolpyruvate carboxykinase. This observation is consistent with the idea that carbon is metabolized through glycolysis, perhaps augmented by catabolism of glycerol, at early times in infection. Late gene expression changes suggest that carbon metabolism shifts toward gluconeogenesis as infection progresses; the glyoxylate cycle genes *ICL1* and *MLS2* were up-regulated, as was the gluconeogenic gene *PCK1*. These changes may reflect increased lipid catabolism for carbon because the β-oxidation gene *FOX2* is up-regulated as well. Interestingly, up-regulation of *ICL1*, *MLS2*, *PCK1*, and *FOX2* occurs upon internalization by macrophages [17]. Our results are consistent with an analysis of green fluorescent protein (GFP) fusion gene expression during infection by Barelle et al. [36], who detected higher expression of *PCK1* and *ICL1* in infecting cells than in glucose-grown cells, which are arguably equivalent to our inoculum. Our interpretation that cells shift from glycoloysis to gluconeogenesis during infection is consistent with the finding by Barelle et al. that a defect in glycolysis blocks establishment of infection, whereas defects in the glyoxylate cycle or gluconeogenesis impair later events in infection [36]. Therefore, the gene expression data reported here are consistent with the observation that glycolysis is required early in infection and that invasive *C*. *albicans* cells respond to phagocytosis at later times during infection.

Our gene expression data correlate with a recent gene expression profile by Chen et al. of whole zebrafish through a time-course of *C*. *albicans* infection [16]. The study normalized samples to the mean expression level over the time-course for each gene. Our early up-regulated genes correlated with genes that Chen et al. detected as up-regulated at 8 hr postinfection (roughly the midpoint of the time-course) and as down-regulated at 0.5 hr postinfection. Because of the normalization procedure, it is reasonable that both of these zebrafish samples would correlate with our early class. Our late up-regulated genes correlate with genes that Chen et al. detected as up-regulated at 16 hr postinfection, close to the end of the time-course. These correlations suggest that *C*. *albicans* carries out similar biological processes in both infection models. Many matches among both early and late genes map to metal ion homeostasis GO terms. This correlation is consistent with the idea that nutritional immunity is used broadly as a defense mechanism [31].

**Fig 9:**
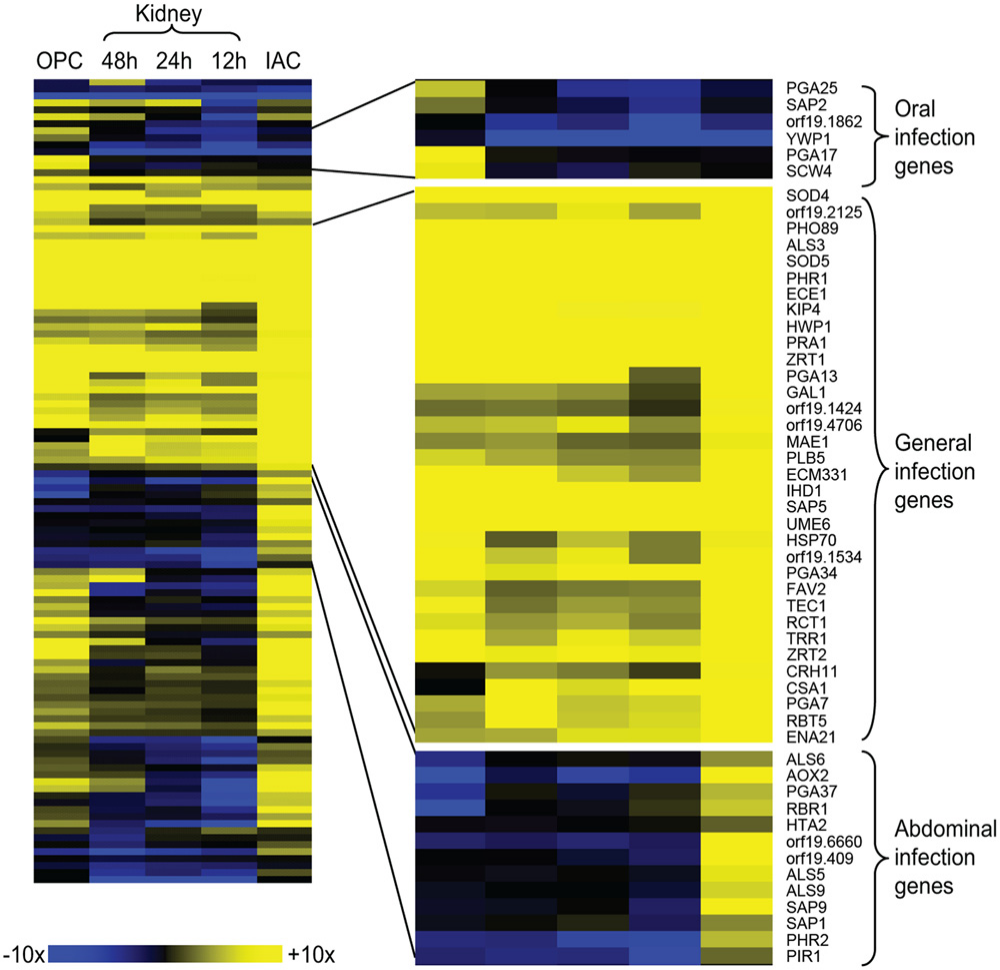
Gene expression during murine infection. Expression levels for 114 genes are compared in three murine infection models: oropharyngeal candidiasis (48 hr postinfection; oropharyngeal candidiasis [OPC]) [13], kidney infection (12, 24, and 48 hr [this study]), and intra-abdominal infection (48 hr postinfection; IAC) [12]. Expression levels are presented as ratios to levels in the inoculum samples used in this study (stationary phase, YPD), and shown as a heat map. Expanded portions illustrate genes induced during oral infection, during all three types of infection, and during abdominal infection. All numerical data for this figure are in S7 Data.

Does *C*. *albicans* express different sets of genes at different infection sites? We can begin to address that question by comparison of the kidney infection profile reported here with infection profiles of other mouse infection models: oropharyngeal candidiasis [13] and abdominal infection [12]. Expression levels for 115 genes were assayed in all three models, and we converted the data to expression ratios relative to the inoculum samples used here (Fig. 9). Thirty-two genes are up-regulated in all infection models, and they include many genes related to hyphal formation (*ALS3*, *ECE1*, *HWP1*, *SOD5*, *TEC1*, *UME6*), zinc limitation (*PRA1*, *ZRT1*, *ZRT2*) and iron limitation (*PGA7*, *RBT5*). Therefore, with the qualification that the dataset is small, there seem to be common driving forces in all three infection models. There are some examples of genes up-regulated only during oral infection, though too few to allow an inference about relevant regulatory or functional relationships. There are also genes up-regulated only during abdominal infection, which include the adhesin genes *ALS5*, *ALS6*, and *ALS9*. These adhesins may be involved in the abscess formation that characterizes this model [12]. The most striking conclusion, though, is that we see no examples of genes that are expressed only during kidney infection. We have sampled only 2% of the *C*. *albicans* genome, and it seems likely that kidney-specific genes will be found. The genes we have sampled are enriched for stress response genes, though, and we speculate that the kidney may be a common target organ for disseminated infection because it is a relatively hospitable environment.

### Gene Regulation during Infection

The *C*. *albicans* transcription factors that govern infection have been extensively studied through both single gene analyses and large scale competitive growth assays [27,37]. We find that strong in vivo expression or up-regulation are excellent predictors of function during invasive infection. Expression level seems to be the most discerning predictor; highly expressed transcription factor genes are much more likely to have measurable impact on infection than the weakly expressed. Such considerations may offer useful prioritization of prospective virulence regulators in pathogens that lack the wealth of prior analysis of *C*. *albicans*.

The infection environment modifies the gene expression impact of the majority of transcription factors that we examined, compared to what is observed under in vitro conditions. The gene expression impact of a mutation in vivo may reflect an amalgam of the direct effects of the transcription factor bound to its target genes, along with input from a spectrum of stress response pathways activated in each mutant when it is unable to proliferate. This explanation seems especially compelling for the *efg1Δ/Δ* mutant. The profile of this strain in vivo displayed a good correlation among down-regulated hyphal genes but little correlation among up-regulated genes, when compared with in vitro *efg1Δ/Δ* expression data [25]. The genes up-regulated in vivo were diverse, with many annotated to the GO term Response to Stress (e.g., *CAP1*, *CAT1*, *HSP104*, *HSP70*, and *YHB1*). Our data suggest that the kidney environment encountered by each mutant is similar, in that host gene expression response is essentially proportional to the fungal burden assessed by fungal RNA level; we see no evidence for qualitative alteration of the host response by individual mutant strains. Therefore, we would expect to see a common set of genes with altered expression in many different attenuated mutants. We see some evidence of a shared gene expression response among all of the mutants assayed. We believe that genome-wide analysis combined with additional replicates, given the low mutant titers, will document a more extensive shared gene expression response among attenuated strains.

Are in vivo mutant profiles informative? In the case of Rim101, comparison of the mutant profiles from two different infection sites offers a simple explanation for niche-specific *rim101Δ/Δ* mutant phenotypes. During invasive growth in the kidney, the *rim101Δ/Δ* mutant is defective in hyphal formation [19]. Our results here provide a simple explanation that was not understood previously (see [14,38] for recent reviews): the *rim101Δ/Δ* mutant has reduced expression of two hyphal regulatory genes, *EFG1* and *TEC1*, during kidney invasion. In contrast, during growth in an abdominal infection model, the *rim101Δ/Δ* mutant is not defective in hyphal formation, nor in *EFG1* or *TEC1* expression [12]. Many mutant strains are attenuated in multiple infection models, and we often infer that the same pathways or functions are thus required for infection in multiple contexts. Interestingly, though, if the gene expression impact of a mutant defect is tailored differently in distinct infection environments, then the same genetic lesion may lead to different causes for attenuation.

If regulatory relationships are modified during infection, then it seems possible that some regulatory pathways may be more prominent during growth in vivo than in vitro. Here, we have identified such a pathway relationship, in which transcription factor Sut1 is required for Zap1 expression, and Zap1 in turn activates zinc acquisition genes that are necessary for proliferation in vivo. The *sut1Δ/Δ* and *zap1Δ/Δ* mutants have very similar gene expression alterations in vivo, an observation that led us to hypothesize that they function in the same pathway. Interestingly, the two mutants have little similarity between their gene expression alterations during growth in vitro, and only the *zap1Δ/Δ* mutant has a zinc acquisition deficiency in vitro. In addition, there is no indication of a functional interrelationship between the orthologous genes in *Saccharomyces cerevisiae*. In other words, the traditionally sought indications that two genes are functionally related were negative. We demonstrated that Sut1 and Zap1 are functionally related in vivo with the demonstration that *ZAP1* overexpression restores zinc acquisition gene expression and virulence to a *sut1Δ/Δ* mutant. This conclusion was strengthened by the finding that overexpression of the zinc transporter gene *ZRT2* also restores virulence to a *sut1Δ/Δ* mutant. Therefore, in vivo profiling data can define biologically relevant functional relationships that are not evident from in vitro analysis.

Our hypothesis is that Rpn4 is functionally related to Sut1 and Zap1, given the similarity of the three mutant gene expression profiles during infection. Rpn4 is not required for zinc acquisition gene expression, so Rpn4 may govern a response to zinc limitation. There are many alternative hypotheses, though, and the issue may be resolved by future functional studies.

### Response to Drug Treatment In Vivo

It seems logical that pharmacological perturbations, like genetic perturbations, may have distinct effects in vivo and in vitro. The gene expression response to the cell wall inhibitor caspofungin is a clear example. The functional spectrum of caspofungin-induced genes seems similar under all conditions and includes many cell wall–modification genes. However, the particular genes that are induced in infecting cells are quite different from those induced in cells grown in vitro. Our finding that a different selection of transcription factor genes is induced by caspofungin in vivo and in vitro helps to explain the overall difference in transcriptional responses.

An unanticipated finding from our caspofungin response profiling is that caspofungin induces many of the same genes that are repressed early in infection. It is not clear why caspofungin reverses the repression of many genes that occurs early in infection; perhaps the high osmolarity of the kidney relieves the need for cell wall reinforcement by eliminating turgor, thus repressing cell wall reinforcing functions early in infection. Alternatively, the sequestration of nutrients in tissue may cause repression of genes early in infection, and perhaps lysis of the first drug-exposed *C*. *albicans* cells relieves nutrient limitation and thus repression. What we find exciting is that the correlation may mean that infecting cells, by virtue of repressing genes that help survive drug treatment, may be in a more drug-susceptible state than in vitro–grown cells. This inference fits well with the study of Wheeler et al. [39], who observed that cell wall β-glucan is more exposed on the *C*. *albicans* cell surface of infecting cells than of in vitro–grown cells. Thus the Wheeler study indicates that cell wall structure is different in vitro and in vivo; our data argue that one source of this difference is the transcriptional regulation of cell wall biogenesis genes.

### Perspective

Many of the key regulatory and environmental signals that have been deduced to govern *C*. *albicans* behavior during infection are indeed manifested at the level of gene expression. Regulatory relationships are modified during infection though, with two main consequences that we have reported. First, some regulatory relationships are more evident in vivo than in vitro, and the in vivo relationships can reveal pathways that are relevant to pathogenicity. Our analysis of *SUT1*, *ZAP1*, and *ZRT2* stands as an example. Second, the response to drug treatment manifested during infection can be distinct from that detected in vitro, as revealed by our analysis of caspofungin treatment. The drug response during infection includes induction of many infection-repressed genes, a relationship that may contribute to drug efficacy. Casadevall and colleagues [40] have argued that virulence is an emergent property, a unique state manifested only when host and pathogen interact. Our results provide support for this concept by illustrating that unique regulatory relationships emerge in the environment of invasive infection.

## Materials and Methods

### Ethics Statement

All animal procedures were approved by the Institutional Animal Care and Use Committee at the Los Angeles Biomedical Research Institute (protocol 011000) and carried out according to the National Institutes of Health (NIH) guidelines for the ethical treatment of animals.

The mice were caged in an AAALAC-accredited facility located on the campus of Harbor-UCLA Research and Education Institute. A full-time veterinarian who specializes in laboratory animal medicine oversaw their care. Caging and husbandry was provided according to the guidelines in the United States Public Health Service publication *Guide for the Care and Use of Laboratory Animals*.

Every attempt was made to treat the mice humanely. The survival and health of the mice was monitored three times daily. Obviously sick, lethargic mice were segregated from the group and euthanized to minimize suffering.

The mice were euthanized by pentobarbital overdose (210 mg/kg), as recommended by the Panel on Euthanasia of the American Veterinary Medical Association.

### Media

*C*. *albicans* strains were grown at 30°C in YPD (2% Bacto peptone, 2% dextrose, 1% yeast extract) or 37°C in RPMI-1640 (with L-glutamine and 0.165M MOPS, without sodium bicarbonate), both with shaking at 200 rpm. Transformants were selected on synthetic medium (2% dextrose, 1.7% Difco yeast nitrogen base with ammonium sulfate and auxotrophic supplements) or on YPD+clonNAT400 (2% Bacto peptone, 2% dextrose, 1% yeast extract, and 400 μg/ml nourseothricin [clonNAT, WERNER BioAgents]) for nourseothricin-resistant isolates. Growth on low-zinc medium [41] was assayed with synthetic medium lacking added zinc (2% dextrose, 1.7% yeast nitrogen base without ammonium sulfate and without zinc sulfate, 0.2% ammonium sulfate, 2.5 μM EDTA, and auxotrophic supplements).

### Construction of pSG1 *IRO1* Plasmid

The *C*. *albicans IRO1* gene was polymerase chain reaction (PCR) amplified from strain SC5314 genomic DNA using primers pSG1 Nde1 IRO1 18 F and pSG1 Nde1 IRO1 257, which have flanking homologous sequences to plasmid pSG1, a derivative of pDDB78 [42] from which the *S*. *cerevisiae TRP1* marker had been deleted. The PCR product was co-transformed with NdeI digested pSG1 into *S*. *cerevisiae* for homologous recombination. The resultant plasmid was isolated from *S*. *cerevisiae* and transformed into *Escherichia coli* for amplification. The plasmid pSG1 *IRO1* can be linearized by Afe1 digestion and then transformed into a His^-^ *C*. *albicans* strain to direct integration at the *IRO1* locus, making the strain His^+^ *IRO1*.

### *C*. *albicans* Strains

*C*. *albicans* deletion mutant strains were constructed in the BWP17 strain background through homologous recombination [43]. Briefly, gene disruption PCR products were synthesized using the plasmids pRS-ARG4 or pGEM-URA3 as templates. The primers were designed to include homology to the sequence immediately upstream of the start codon or the sequence immediately following the stop codon of the target gene. Arg^+^ Ura^+^ homozygous deletion strains were then made His^+^ along with restoring *IRO1* by integration of linearized plasmid pSG1-*IRO1* at the *HIS1* locus. For complementation of deletion strains, PCR primers were designed to amplify genomic DNA of strain SC5314 from 1 kb upstream to 0.5 kb downstream of the open reading frame of specific genes. Shorter distances were used when there were additional genes located within this region. These primers have 5′ flanking sequences with homology to pSG1 *IRO1*. The resulting PCR product was co-transformed into *S*. *cerevisiae* with Not1 and Sac1 digested pSG1 *IRO1*. Plasmid DNA was isolated, transformed into *E*. *coli*, and isolated plasmid DNA was digested with AfeI and transformed into the respective *C*. *albicans* mutant strains. The complementing cassette was targeted to the *IRO1* locus and these complemented strains were then also His^+^ *IRO1*.

Where indicated, we used the prototrophic reference strain CW696 as a control. It is derived from the SC5314 background [44] strain DAY286 [45], which in turn derives from strain BWP17 [43], as do all of the deletion mutants studied here. Strain DAY286 was transformed with Afe1-digested plasmid pSG1 *IRO1* to restore both *HIS1* and *IRO1*. His+ transformants were selected on CSM-his. We verified that CW696 expresses *IRO1* through nanoString assays, and confirmed that its virulence is comparable to that of strain SC5314, based on median survival assays in the mouse hematogenously disseminated infection model.

For *ZAP1* and *ZRT2* overexpression strains, PCR primers were designed to amplify the NAT1-pTDH3 cassette from plasmid CJN542 [15]. These primers were designed to include homology to the sequence immediately upstream of the start codon or the sequence immediately following the stop codon of the *ZAP1/ZRT2* gene. The PCR products were transformed into the respective *C*. *albicans* mutant strains, replacing one allele of *ZAP1/ZRT2* promoter with the *TDH3* promoter by homologous recombination. *C*. *albicans* strains used in this study are listed in S1 Table. PCR primers used in this study are listed in S2 Table.

### Mouse Infection and Survival Assay

Male Balb/c mice weighing 20–22g (Taconic Farms) were used for all studies. In the survival studies, five mice per strain of C. *albicans* were inoculated intravenously with 5 × 10^5^ yeast-phase cells that had been grown in YPD at 30°C to saturation as described previously [46]. A portion of each inoculum was plated on a YPD plate, and CFU was measured after 2 d of growth to check viability of cells in the inoculum. The animals were monitored three times daily for 21 d, and moribund mice were humanely euthanized. The results of the survival experiments were analyzed with the Log-Rank Test. For isolation of host and fungal RNA and histopathology, three mice per strain were inoculated intravenously with 1 × 10^6^ yeast-phase organisms, and the kidneys were harvested at specific time points. The right kidney was snap frozen in liquid nitrogen and stored at −80°C for later RNA extraction. The left kidney ways fixed in zinc-buffered formalin, embedded in paraffin, sectioned, and stained with Periodic acid-Schiff. All mouse experiments were approved by the Animal Care and Use Committee at the Los Angeles Biomedical Research Institute (protocol 011000) and carried out according to the National Institutes of Health (NIH) guidelines for the ethical treatment of animals.

### RNA Isolation from Mouse Tissues

RNA isolations were performed using Qiagen RNeasy mini kit (Cat#74104) with modifications described here. Approximately 1.5 ml of buffer RLT with 1% β-ME was added to each kidney immediately before homogenization using gentelMACS dissociator (Miltenyi Biotec) on pre-loaded setting RNA_02.01. The M tube (Miltenyi Biotec) was centrifuged at 2,000 rpm for 1 min in Eppendorf tabletop centrifuge 5810R at room temp. 600 μl homogenate was transferred to a fresh 2 ml screw-cap tube and mixed with 600 μl Phenol:Chloroform:Isoamyl Alcohol 25:24:1 and 300 μl of Zirconia beads (Ambion), vortexed on a mini-beadbeater (Biospec Products) for 3 min, and centrifuged at 14,000 rpm for 5 min in a 4°C cold room. The aqueous phase was transferred to a new 1.5 ml microfuge tube, mixed well with equal volume of 70% ethanol, and loaded onto the RNeasy spin column. The washing and RNA elution steps were carried out following Qiagen Quick-Start Protocol for RNeasy Mini Kit (Cat#74104).

### Caspofungin Treatment

For caspofungin treatment in vivo, caspofungin was administrated at a dose of 1 mg/kg(approximately 20 ug per mouse) intraperitoneally at 24 hr postinfection. The control mice received a similar volume of PBS intraperitoneally. The mice were killed 2 hr after drug administration and the kidneys were collected for RNA isolation. For caspofungin treatment in vitro, an overnight YPD 30°C culture of wild-type SC5314 was diluted 1:1,000 into 50 ml RPMI cultures and grown at 37°C with 200 rpm shaking for 4 hr. Caspofungin was then added to a final concentration of 100 ng/ml, and the control cultures received equal amount of water. Cells were collected by filtration 2 hr after drug administration.

### NanoString Codeset Design

Because nanoString technology is not genome-wide, we developed strategies to select high-priority fungal and host genes for investigation. First, we selected 248 *C*. *albicans* genes that play important roles in environmental responses, such as iron/zinc acquisition, oxidative/nitrosative stress, pH response, hyphal growth, and general stress responses. We reasoned that these environmental response gene probes (ER codeset) should give us clues about what environmental challenges *Candida* cells face during infection and which pathways are employed to cope with such challenges. Secondly, we designed a codeset to include 231 *Candida* genes that specify known and predicted transcription factors (TF codeset). Finally, we selected 46 mouse genes that are critical in host responses (HR codeset) to microbial infection, including genes encoding for fungal pattern recognition receptors, chemokines and chemokine receptors, interleukins and interleukin receptors, interferons and antimicrobial peptides. All the *Candida* gene codesets also include control genes TDH3 and YRA1, and the mouse gene codeset includes control genes ACTB, GAPDH, and PPIA. The complete list of genes for all codesets is shown in S1, S3, and S4 Data.

### NanoString Sample Preparation and Data Analysis

For each nanoString assay, 10 μg of total tissue RNA isolated from a mouse kidney or 100 ng of pure *Candida* RNA isolated from an in vitro culture was mixed with a nanoString codeset mix and incubated at 65°C overnight (16–18 hr). The reaction mixes were loaded on the nanoString nCounter Prep Station for binding and washing, and the resultant cartridge was transferred to the nanoString nCounter digital analyzer for scanning and data collection. A total of 600 fields were captured per sample. The raw data were first adjusted for binding efficiency and background subtraction, following nCounter data analysis guidelines. The total adjusted counts (i.e., before normalization using the internal control genes) for genes on the same codeset were used to estimate relative fungal RNA abundance in each sample. For example, 10 ug RNA isolated from kidney 24 hr postinfection by wild-type strain SC5314 generated 324,648 total counts for 150 genes (mean of six determinations), while that from *rim101Δ/Δ* infected kidney generated 49,083 counts (mean of three determinations). We estimated that at 24 hr postinfection, the relative fungal RNA abundance for *rim101Δ/Δ* infected kidney is roughly 15% (49,083/324,648) of that of the wild type. Following the same logic, we estimated that fungal RNA consists approximately 0.1% of total RNA isolated from kidney infected by the wild-type strain SC5314 at the 24 hr time point (by comparing to total counts from in vitro samples).

To calculate gene expression ratios among different samples, we normalized adjusted raw counts using internal control genes. For the ER codeset, data were normalized using the control gene *TDH3*. For the TF codeset, data were normalized by total counts of 231 genes. We tested a number of methods for normalization, including using another control gene *YRA1*, using total counts for all genes, and using geometric mean of robustly expressed genes. We found that the normalization factors resulting from these methods are similar and will not affect our main conclusions should we use a different method for normalization. For the HR codeset, data were normalized using the geometric mean of three internal control genes: ACTB, GAPDH, and PPIA. All expression ratios were calculated using mean values of three independent biological samples, and statistical significance was determined by two-tailed Student’s *t*-test (*n* = 3, *p* < 0.05, unless specified otherwise). The heat maps were generated using Multiexperimental Viewer 4.9.0. Dataset comparisons were carried out with one-sided Fisher's Exact Test, using query gene sets from in vivo nanoString data described here (applying cut-off at 2X, 4X or 10X changes) and the entire set of nanoString probes as a background set. Queries were matched to a database we assembled of 166 published expression datasets, as well as nanoString datasets we generated during this study.

### Quantitative Reverse Transcription PCR

Quantitative reverse transcription PCR reactions were carried out as previously described [47]. Briefly, 10 μg total RNA was treated with the DNA-free kit (Ambion) followed by first-strand cDNA synthesis from half of the DNA-free RNA using the AffinityScript multiple temperature cDNA synthesis kit (Stratagene). Absence of DNA contamination was confirmed using control sets for which reverse transcriptase was omitted from the cDNA reaction. Primers were designed to amplify a 150–200 bp region for target genes including *TDH3*, which was used as a reference gene for normalization. 2X iQ SYBR Green Supermix (Bio-Rad), 1 μl of first-strand cDNA reaction mixture, and 0.1 μM of primers were mixed in a total volume of 50μl per reaction. Real-time PCR was performed in triplicate using an iCycler iQ real-time PCR detection system (Bio-Rad). The program for amplification had an initial denaturation step at 95°C for 5 min, followed by 40 cycles of 95°C for 45 s and 58°C for 30 s. Product amplification was detected using SYBR Green fluorescence during the 58°C step, and specificity of the reaction was monitored by melt-curve analysis following the real-time program. Gene expression was determined using Bio-Rad iQ5 software (ΔΔ*CT* method).

## Supporting Information

S1 Data Expression of *C*. *albicans* environmentally responsive genes during infection. NanoString pobes for 248 *C*. *albicans* environmental responsive genes were chosen based on published gene expression data and preliminary data generated in this study, as indicated by the PubMed identification (PMID) number in the first row in the tab "probe selection." Three independent biological samples at each time point (0 hr/inoculum, 12 hr, 24 hr, and 48 hr postinfection) were used to determine gene expression levels by nanoString n-counter. The expression data were normalized using the internal control gene TDH3. Expression ratios were calculated using mean values of three independent biological samples, and statistical significance was determined by two-tailed Student’s *t*-test (*n* = 3, *p* < 0.05).
(XLSX)

S2 Data Comparison of *C*. *albicans* environmental response gene expression during invasive infection to other profiling datasets. Dataset comparisons were carried out with one-sided Fisher's Exact Test, using query gene sets from in vivo nanoString profiling data in the current study (applying cutoff at 2X, 4X, or 10X changes), and the entire set of nanoString probes (i.e., 248 environmental response genes) as a background set. Queries were matched to a database we assembled from 166 published expression datasets (PMID indicated in the second row), as well as datasets generated in this study (labeled "this study").
(XLSX)

S3 Data Expression of mouse immune response genes during *Candida* infection. For mice infected with wild-type (SC5314) *Candida albicans*, expression level for 46 mouse immune response genes were determined by nanoString from three independent biological samples at each time point (0 hr/uninfected, 12 hr, 24 hr and 48 hr postinfection kidney). All host response datasets were normalized using the geometric mean of three internal control genes ACTB, GAPDH, and PPIA. Expression ratios were calculated using mean values of three independent biological samples, and statistical significance was determined by two-tailed Student’s *t*-test (*n* = 3, *p* < 0.05). For mice infected with transcription factor mutants (*n* = 3) and respective complemented strains (*n* = 2 or 3), expression level for 46 mouse immune response genes were determined by nanoString at 24 hr postinfection.
(XLSX)

S4 Data Expression of *C*. *albicans* transcription factor genes during infection. Expression level for 231 *C*. *albicans* transcription factor genes were determined by nanoString from three independent biological samples at each time point (0 hr/inoculum, 12 hr, 24 hr, and 48 hr postinfection kidney). Expression ratios were calculated using mean values of three independent biological samples, and statistical significance was determined by two-tailed Student’s *t*-test (*n* = 3, *p* < 0.05).
(XLSX)

S5 Data Expression profiling of *C*. *albicans* transcription factor mutants during invasive infection. Expression level for 148 *C*. *albicans* environmental response genes were determined by nanoString from three independent biological samples for each mutant strain (and at least two independent samples for each complemented strain) at 24 hr postinfection, except for the efg1 mutant and complement, which were determined at 48 hr postinfection (due to the extremely low fungal burden at 24 hr postinfection). Expression ratios were calculated using mean values of independent biological samples, and statistical significance was determined by two-tailed Student’s *t*-test (*n* = 3, *p* < 0.05). For the rim101 mutant and complement strains (see tab "rim101"), expression profiles of 144 environmental response genes under two infection models and one in vitro growth condition were compared to demonstrate niche specific gene expression regulation. In the "TF mutants summary" tab, gene expression ratio to the wild type, *p*-value, and gene function based on published data were listed to simplify cross-referencing.
(XLSX)

S6 Data *C*. *albicans* response to caspofungin treatment in vivo and in vitro. Expression level for 248 *C*. *albicans* environmental response genes and 231 transcription factor genes were determined under in vivo (invasive infection/kidney) and in vitro (RPMI) conditions, with or without caspofungin treatment. Mean expression levels of each gene from three independent biological samples were used to calculate the ratio between the caspofungin treated versus untreated samples, and statistical significance was determined by two-tailed Student’s *t*-test (*n* = 3, *p* < 0.05). The early response data (12 hr postinfection) from S1 Data are included here for comparison.
(XLSX)

S7 Data Numerical values for all figures. (XLS)

S1 Fig Raw *C*. *albicans* gene expression data from infected tissue. NanoString probe counts for infected (pink and blue data points) and uninfected (yellow data points) kidney samples are presented as scatter plots for 12, 24, and 48 hr postinfection (panels A, B, and C, respectively).
(PDF)

S2 Fig QRT-PCR confirmation of nanoString results for selected genes. (PDF)

S3 Fig Complementation of transcription factor mutant gene expression alterations. Each plot shows the fold-change for expression of 148 environmental response genes in mutant versus wild type (*x*-axis) and mutant versus complemented strain (*y*-axis) during infection. Numerical data may be found in S5 Data. Complete complementation yields all points on a diagonal and a slope of 1. Mutants and complemented strains shown are for the genes (A) *RIM101* at 24 hr postinfection, (B) *EFG1* at 48 hr postinfection, (C) *ROB1* at 24 hr postinfection, (D) *ZAP1* at 24 hr postinfection, (E) *RPN4* at 24 hr postinfection, and (F) *SUT1* at 24 hr postinfection.
(PDF)

S4 Fig (A). Growth of *rpn4Δ/Δ*, *sut1Δ/Δ*, and *zap1Δ/Δ* mutants, as well as respective complemented strains, in low zinc medium [41], plotted as A_600_ versus time. (B). Nanostring expression determinations for 148 environmental response genes in the *rpn4Δ/Δ*, *sut1Δ/Δ*, and *zap1Δ/Δ* mutants, relative to wild type, at 24 hr postinfection (left) and during growth in RPMI at 37°C (right), are displayed in heat map representations.
(PDF)

S1 Table *C*. *albicans* strains. (DOC)

S2 Table Synthetic oligonucleotides. (DOCX)

## Abbreviations

FET: Fisher's exact test
**GO**: **Gene Ontology**
HDC: hematogenously disseminated candidiasis
hr: hours
IAC: intra-abdominal candidiasis
NOX: NADPH oxidase 1
OPC: oropharyngeal candidiasis
PCR: polymerase chain reaction
PMID: PubMed Identification
PI: postinfection
QRT-PCR: quantitative reverse transcription polymerase chain reaction
RPMI: Roswell Park Memorial Institute medium 1640
WT: wild type
YPD: yeast extract peptone dextrose medium
