# Supplementary figures and images for: Activation and Alliance of Regulatory Pathways in C. albicans during Mammalian Infection
Source: PLoS Biol. 2015 Feb 18;13(2):e1002076. doi: 10.1371/journal.pbio.1002076 (PMC4333574; doi:10.1371/journal.pbio.1002076)

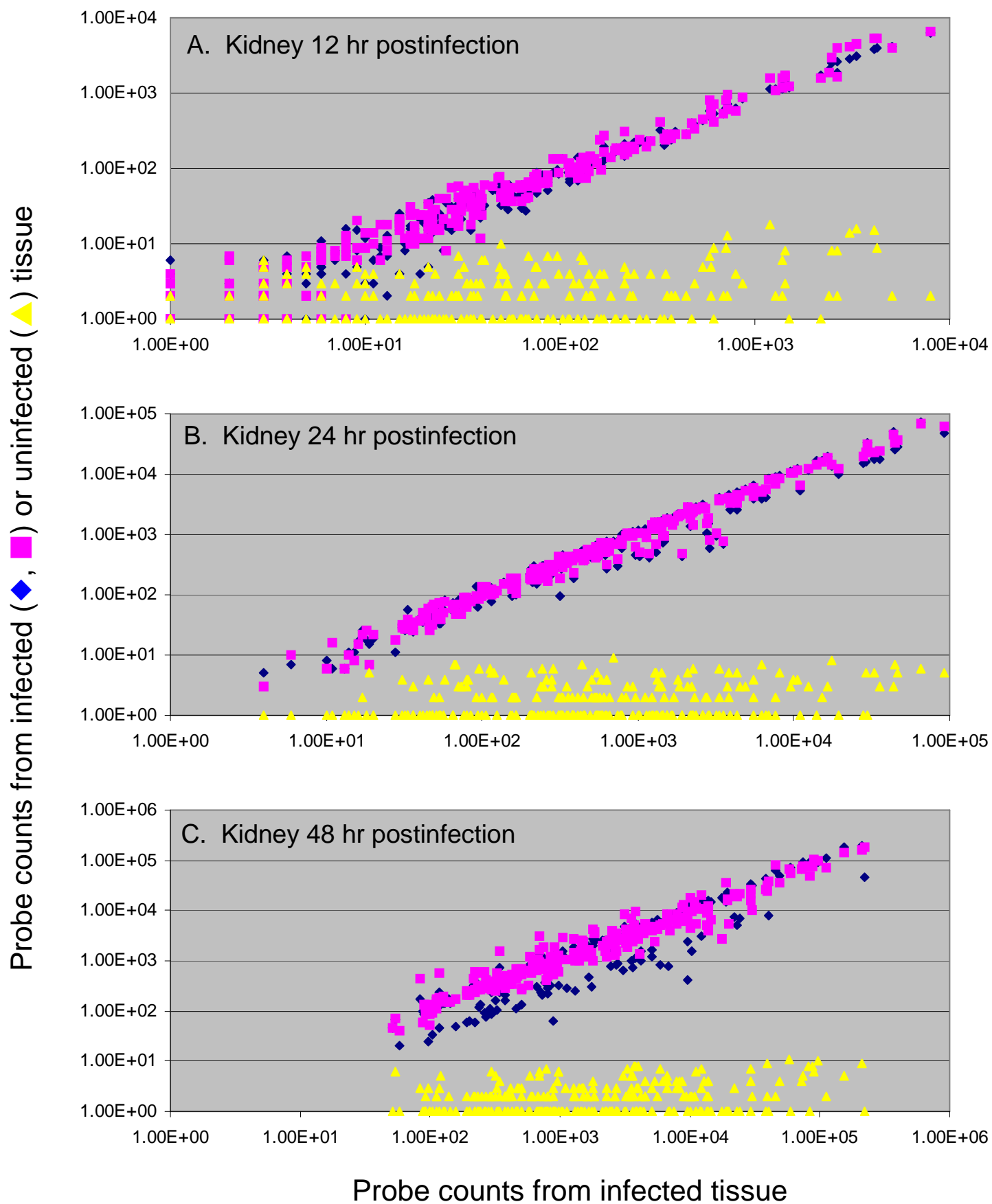

Supplement: S1 Fig — NanoString probe counts for infected (pink and blue data points) and uninfected (yellow data points) kidney samples are presented as scatter plots for 12, 24, and 48 hr postinfection (panels A, B, and C, respectively). (PDF) [file pbio.1002076.s008.pdf]

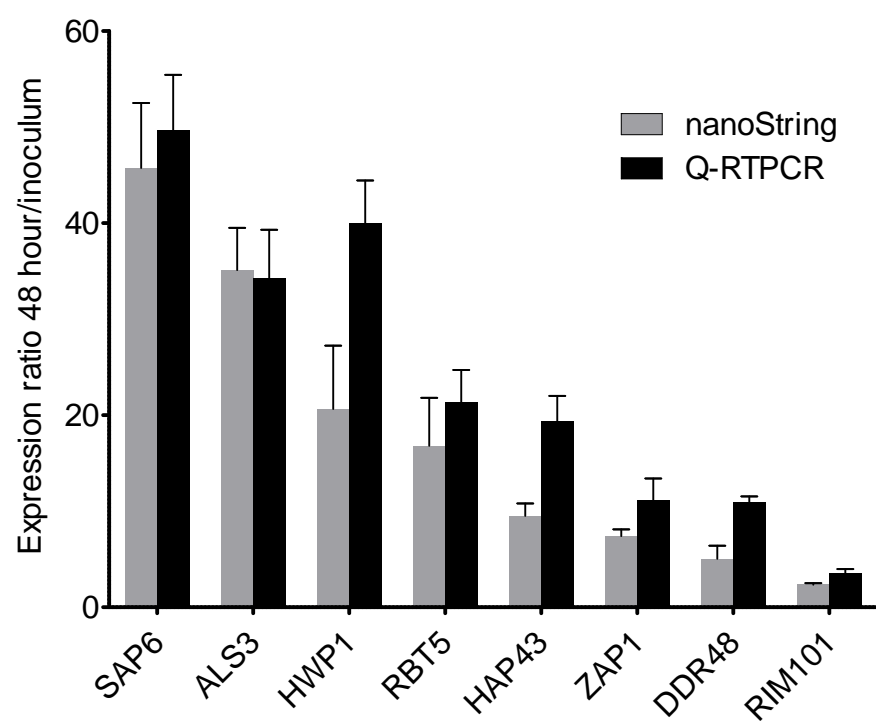

Supplement: S2 Fig — (PDF) [file pbio.1002076.s009.pdf]

A

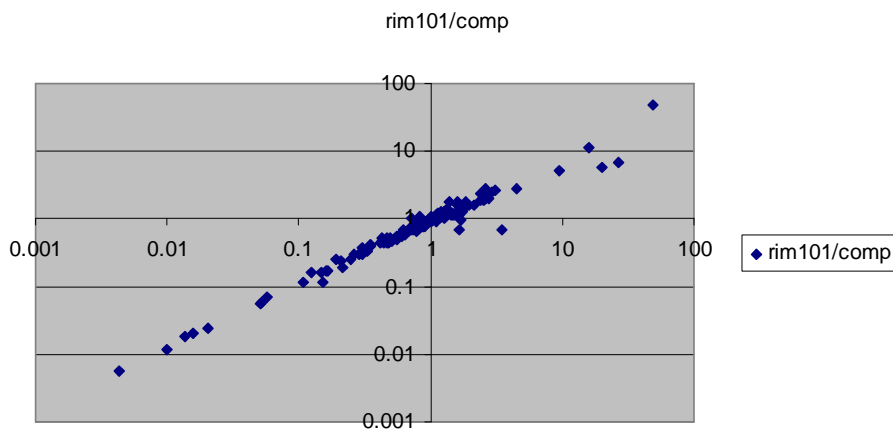

B

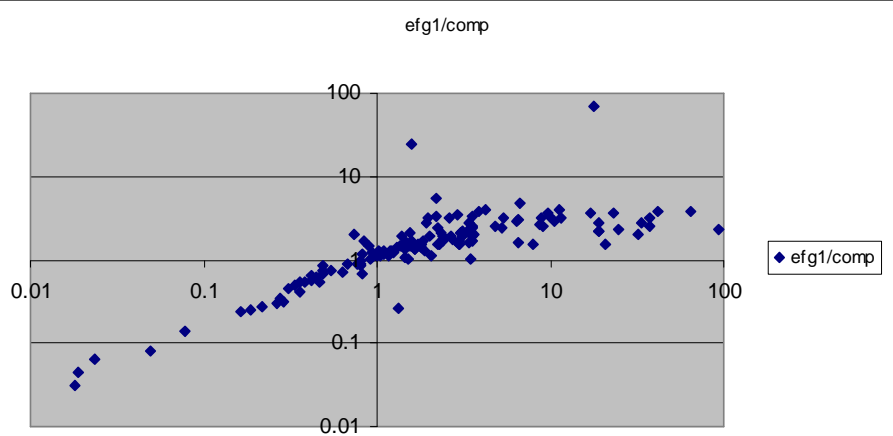

C

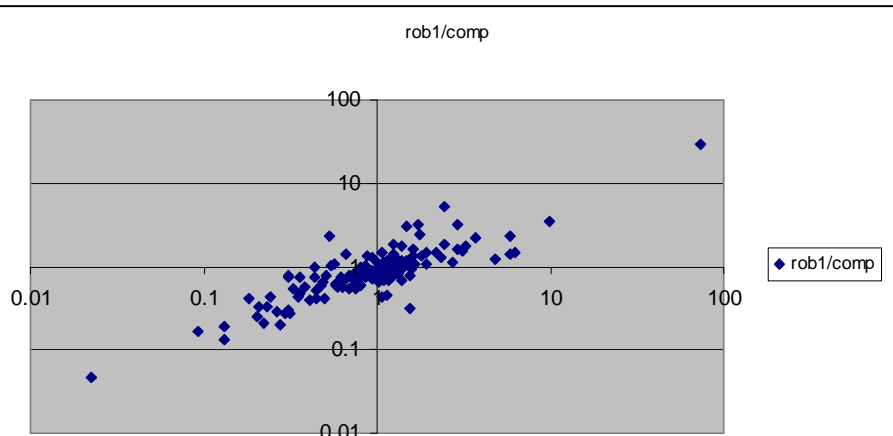

D

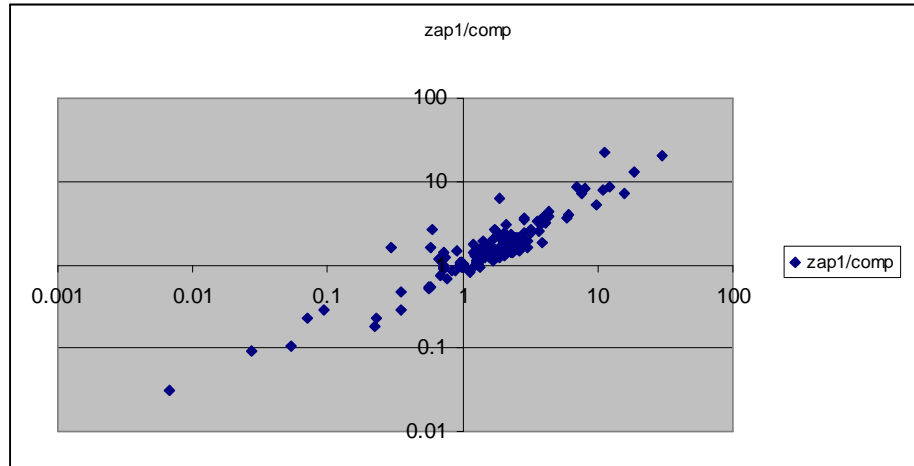

E

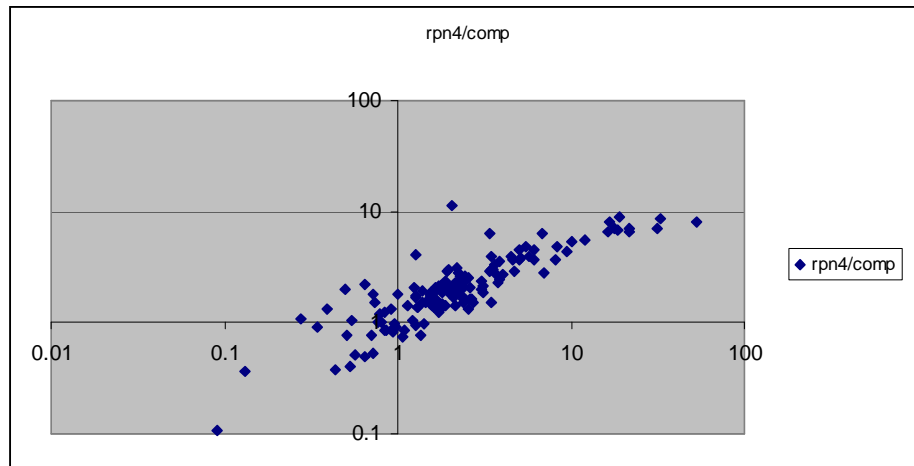

F

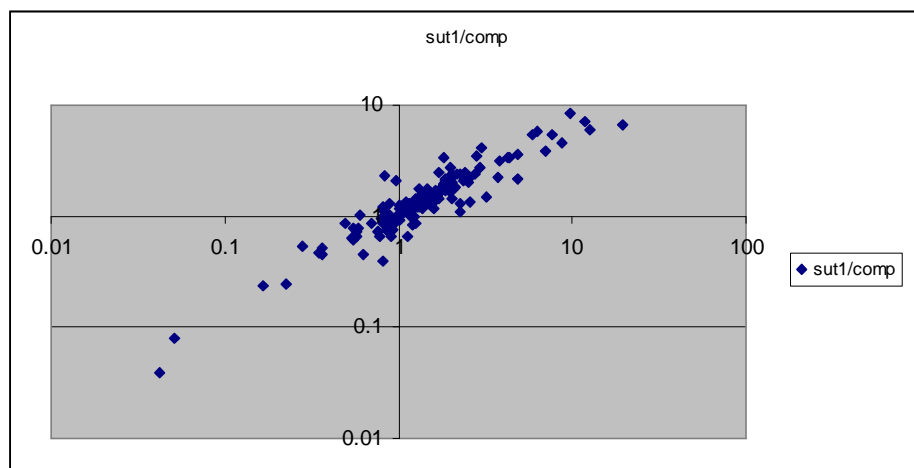

Supplement: S3 Fig — Each plot shows the fold-change for expression of 148 environmental response genes in mutant versus wild type (x-axis) and mutant versus complemented strain (y-axis) during infection. Numerical data may be found in S5 Data. Complete complementation yields all points on a diagonal and a slope of 1. Mutants and complemented strains shown are for the genes (A) RIM101 at 24 hr postinfection, (B) EFG1 at 48 hr postinfection, (C) ROB1 at 24 hr postinfection, (D) ZAP1 at 24 hr postinfection, (E) RPN4 at 24 hr postinfection, and (F) SUT1 at 24 hr postinfection. (PDF) [file pbio.1002076.s010.pdf]

A

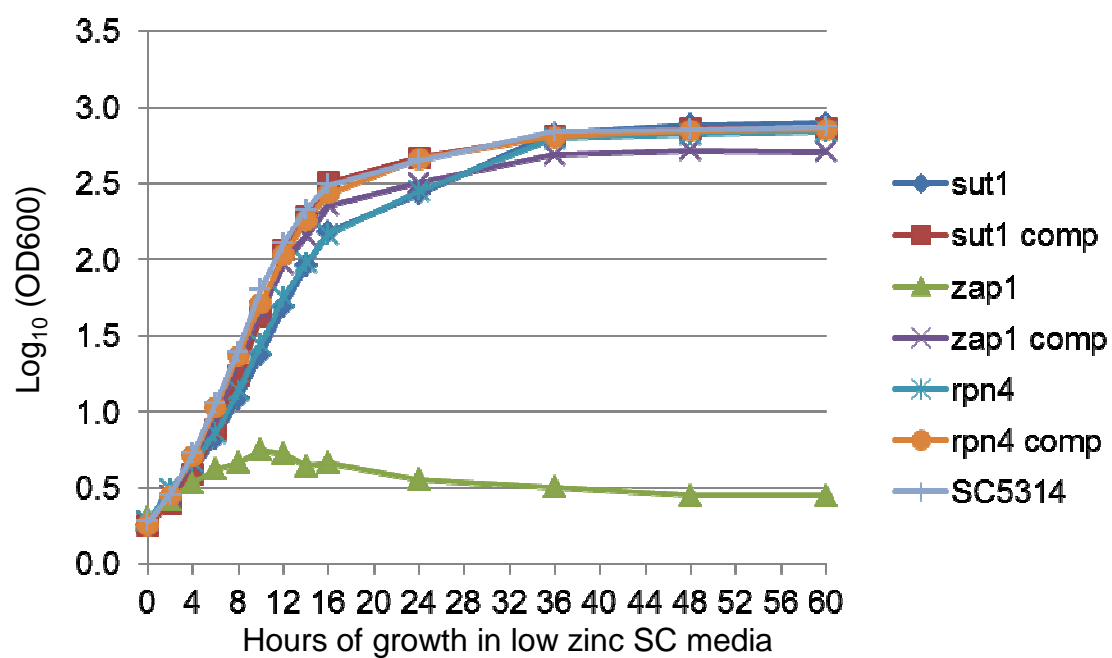

B

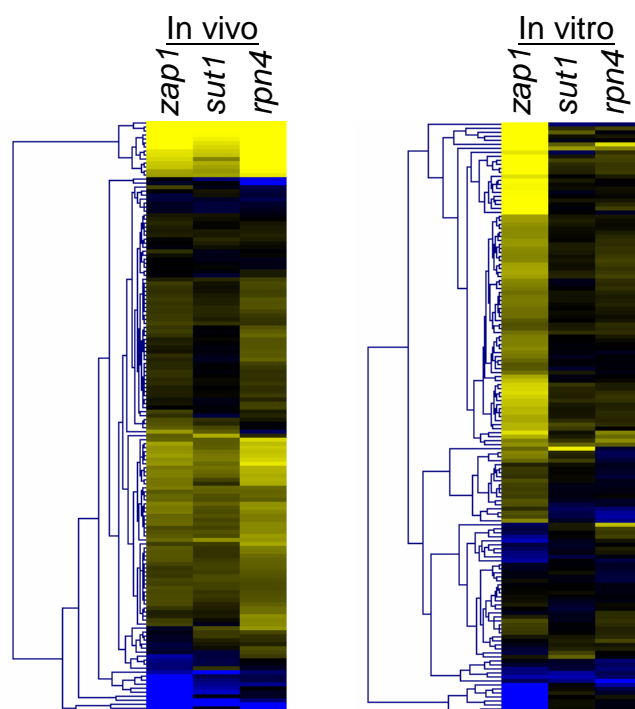

Supplement: S4 Fig — (B). Nanostring expression determinations for 148 environmental response genes in the rpn4Δ/Δ, sut1Δ/Δ, and zap1Δ/Δ mutants, relative to wild type, at 24 hr postinfection (left) and during growth in RPMI at 37°C (right), are displayed in heat map representations. (PDF) [file pbio.1002076.s011.pdf]
