# Supplementary material for: Activation and Alliance of Regulatory Pathways in C. albicans during Mammalian Infection
Source: PLoS Biol. 2015 Feb 18;13(2):e1002076. doi: 10.1371/journal.pbio.1002076 (PMC4333574; doi:10.1371/journal.pbio.1002076)
Supplement: S1 Table — (DOC) [file pbio.1002076.s012.doc]

Supplemental Table 1. List of *Candida albicans* strains.

| Strain | Genotype | Reference |
| --- | --- | --- |
| SC5314 | Wild type | [1] |
| BWP17 | *ura3Δ iro1Δ::λimm434 arg4::hisG his1::hisG*  *ura3Δ iro1Δ::λimm434 arg4::hisG his1::hisG* | [2] |
| DAY25 | *ura3Δ iro1Δ::λimm434 arg4::hisG HIS1::hisG rim101::ARG4*  *ura3Δ iro1Δ::λimm434 arg4::hisG his1::hisG rim101::URA3* | [3] |
| DAY44 | *ura3Δ iro1Δ::λimm434 arg4::hisG HIS1::hisG rim101::ARG4::pRIM101::HIS1*  *ura3Δ iro1Δ::λimm434 arg4::hisG his1::hisG rim101::URA3* | [3] |
| DAY286 | *ura3∆ iro1Δ::λimm434 ARG4:URA3:arg4::hisG his1::hisG*  *ura3∆ iro1Δ::λimm434 arg4::hisG his1::hisG* | [4] |
| CW696 | *ura3Δ iro1Δ::pHIS1 IRO1::λimm434 ARG4:URA3:arg4::hisG his1::hisG*  *ura3Δ iro1Δ::λimm434 arg4::hisG his1::hisG* | This study |
| CW704 | *ura3Δ iro1Δ::pHIS1 IRO1::λimm434 arg4::hisG his1::hisG sut1::ARG4*  *ura3Δ iro1Δ::λimm434 arg4::hisG his1::hisG sut::URA3* | This study |
| CW730 | *ura3Δ iro1Δ::pHIS1 IRO1::λimm434 arg4::hisG his1::hisG rob1::ARG4*  *ura3Δ iro1Δ::λimm434 arg4::hisG his1::hisG rob1::URA3* | This study |
| CW756 | *ura3Δ iro1Δ::pHIS1 IRO1::λimm434 arg4::hisG his1::hisG zap1::ARG4*  *ura3Δ iro1Δ::λimm434 arg4::hisG his1::hisG zap1::URA3* | This study |
| CW785 | *ura3Δ iro1Δ::pHIS1 IRO1::λimm434 arg4::hisG his1::hisG ROB1 rob1::ARG4*  *ura3Δ iro1Δ::λimm434 arg4::hisG his1::hisG rob1::URA3* | This study |
| CW792 | *ura3Δ iro1Δ::pHIS1 IRO1::λimm434 arg4::hisG his1::hisG rpn4::ARG4*  *ura3Δ iro1Δ::λimm434 arg4::hisG his1::hisG rpn4::URA3* | This study |
| CW886 | *ura3Δ iro1Δ::pHIS1 RPN4 IRO1::λimm434 arg4::hisG his1::hisG rpn4::ARG4*  *ura3Δ iro1Δ::λimm434 arg4::hisG his1::hisG rpn4::URA3* | This study |
| CW894 | *ura3Δ iro1Δ::pHIS1 ZAP1 IRO1::λimm434 arg4::hisG his1::hisG zap1::ARG4*  *ura3Δ iro1Δ::λimm434 arg4::hisG his1::hisG zap1::URA3* | This study |
| CW1018 | *ura3Δ iro1Δ::pHIS1 IRO1::λimm434 arg4::hisG his1::hisG efg1::ARG4*  *ura3Δ iro1Δ::λimm434 arg4::hisG his1::hisG efg1::URA3* | This study |
| CW1020 | *ura3Δ iro1Δ::pHIS1 EFG1 IRO1::λimm434 arg4::hisG his1::hisG efg1::ARG4*  *ura3Δ iro1Δ::λimm434 arg4::hisG his1::hisG efg1::URA3* | This study |
| CW1035 | *ura3Δ iro1Δ::pHIS1 SUT1 IRO1::λimm434 arg4::hisG his1::hisG sut1::ARG4*  *ura3Δ iro1Δ::λimm434 arg4::hisG his1::hisG sut1::URA3* | This study |
| WX102 | *ura3Δ iro1Δ::pHIS1 IRO1::λimm434 arg4::hisG his1::hisG sut1::ARG4 pAgTEF1-NAT1-AgTEF1UTR-TDH3-ZAP1*  *ura3Δ iro1Δ::λimm434 arg4::hisG his1::hisG sut::URA3 ZAP1* | This study |
| WX106 | *ura3Δ iro1Δ::pHIS1 IRO1::λimm434 arg4::hisG his1::hisG rob1::ARG4 pAgTEF1-NAT1-AgTEF1UTR-TDH3-ZAP1*  *ura3Δ iro1Δ::λimm434 arg4::hisG his1::hisG rob1::URA3 ZAP1* | This study |
| WX134 | *pAgTEF1-NAT1-AgTEF1UTR-TDH3-ZAP1* (SC5314)  *ZAP1* | This study |
| WX137 | *pAgTEF1-NAT1-AgTEF1UTR-TDH3-ZRT2* (SC5314)  *ZRT2* | This study |
| WX144 | *ura3Δ iro1Δ::pHIS1 IRO1::λimm434 arg4::hisG his1::hisG sut1::ARG4 pAgTEF1-NAT1-AgTEF1UTR-TDH3-ZRT2*  *ura3Δ iro1Δ::λimm434 arg4::hisG his1::hisG sut::URA3 ZRT2* | This study |

References:

1. Gillum AM, Tsay EY, Kirsch DR (1984) Isolation of the Candida albicans gene for orotidine-5'-phosphate decarboxylase by complementation of S. cerevisiae ura3 and E. coli pyrF mutations. Mol Gen Genet 198: 179-182.

2. Wilson RB, Davis D, Mitchell AP (1999) Rapid hypothesis testing with Candida albicans through gene disruption with short homology regions. J Bacteriol 181: 1868-1874.

3. Davis D, Edwards JE, Jr., Mitchell AP, Ibrahim AS (2000) Candida albicans RIM101 pH response pathway is required for host-pathogen interactions. Infect Immun 68: 5953-5959.

4. Davis DA, Bruno VM, Loza L, Filler SG, Mitchell AP (2002) Candida albicans Mds3p, a conserved regulator of pH responses and virulence identified through insertional mutagenesis. Genetics 162: 1573-1581.
