# Supplementary material for: Activation and Alliance of Regulatory Pathways in C. albicans during Mammalian Infection
Source: PLoS Biol. 2015 Feb 18;13(2):e1002076. doi: 10.1371/journal.pbio.1002076 (PMC4333574; doi:10.1371/journal.pbio.1002076)
Supplement: S2 Table — (DOCX) [file pbio.1002076.s013.docx]

Supplemental Table 2. List of primers.

| Primer name | Sequence 5’-3’ |
| --- | --- |
| pSG1 NdeI IRO1_18_F | CGAAGGAAGGAGCACAGACTTAGATTGGTAAATCGGAAACAATCAAAAGAAAG |
| pSG1 NdeI IRO1_257down_R | GTTAAGAATACTGGGCAATTTCATGTTTCTTGCAGGAAATAAGATTGCAAAA |
| ZAP1_del_F | GTAATGCCGTGTGGTGTTACTTGGTAGCACTTTGATCATCTATACACGACGTATCTTAATTTAGAAGTAAATCCCTTTACAATAACAACAATAAGAAGAAGTTTCCCAGTCACGACGTT |
| ZAP1_del_R | CGAAACAACATATTAGCACATTTTTGAAAATATTACATTATCGACAGTGTCTTTGATTGCTATGAATGTACACCTCTCCTCTTTAGTCATCTCATTTACTCGTGGAATTGTGAGCGGATA |
| ROB1_del1_Fornew | TAGAAATAAACTTAGAAACCAAAAAAAAAACACGAGAAGTCTAAAACTAAAAAAAAAATATAAAGAACGATTCTGAACTATTAGAACATTCAGGAACACCTTTCCCAGTCACGACGTT |
| ROB1_del1_Revnew | TCATTCTATGTATATATAATGTTAATCTTCATATAATTGAAAAAACTATTTAGAATTTAAAATAAAAAACAAAAAATAAAAACTAGTAATAATAATAACTGTGGAATTGTGAGCGGATA |
| ROB1_del2_Fnew | ATGACACCAAGTTCAACTAAAAAAATTAAGCAAAGAAGAAGTACTTCGTATGTATTTATATGATTGTTACTTTTTTTGGATTAATAGGAGATTACTAACATTTCCCAGTCACGACGTT |
| ROB1_del2_Rnew | TTATAAACGAAATCCTTCTGTATCAAAACTAAAAATATTATTAAATGTAGATCCATTGTTATCTACTATAGAATTAAAAAATGTTGGATCTAATATTTCTGTGGAATTGTGAGCGGATA |
| RPN4_del 1_for | ACGTTTTTACTTTTAAACTTACACATTTTATTAAATTCTTAATACCCATTAGATGACTTCATTAGCTATATTACCACAGTTAAAGAGAACCATCACTGACTTTCCCAGTCACGACGTT |
| RPN4_del 1_rev | TGAAATCTAAAATAATAAACCCCCCTATAGTTTGCATTAAAATGAAAAAATGCTGAAACCTTCAAAAGATAAAAGTAAACTAATATCCTTTCGTGGAATTGTGAGCGGATA |
| RPN4_del 2_for | ATGACTTCATTAGCTATATTACCACAGTTAAAGAGAACCATCACTGACATTATGGACGAAGAATTATATCAATCACCTTCGTCTCCAAATTCTATGACGATTTCCCAGTCACGACGTT |
| RPN4_del 2_rev | TTATACTGGAATATATTCAACATTCTCTTTAGCTTCATTGATTAAATCTAATGCGTCTTGTCCAACTAAACCATGCTTGATTTTGATGTGTCTTGATAAGGTGGAATTGTGAGCGGATA |
| SUT1_del 1_for | CTATTTCTTATTTCTTTTTCATTCAATATAGATTATACTCATTATTATTATTTATTCACCTTTCCCAGTCACGACGTT |
| SUT1_del 1_rev | ATGTATATATAATAAATGCAAAAATTAAAAAAACAAAACCCTCTAACTTTAACTCTAACTGTGGAATTGTGAGCGGATA |
| SUT1_del 2_for | ATGTCTGACTTATTATCTTATTCCATTATGAATAACACTTCCAAATATCATCATTCAAATTTTCCCAGTCACGACGTT |
| SUT1_del 2_rev | CTAATGTTTTTGTAATTTCTTTTTCATAATTTTTTTAGCACCACCAGTAGTGGTAGTAGTGTGGAATTGTGAGCGGATA |
| Comp_ZAP1_For | TTCACACAGGAAACAGCTATGACCATGATTACGCCAAGCTTTACCTTTACTAAGTTGTCAAGAAGTGC |
| pSG1-compZAP1_rev | CCATTAACTTTAAACCATCTTCGACCGTCATGTCCTTTCTTTTTAGCCAAAGTGTAAGTTGTAAGAGA |
| Comp_RPN4_For | TTCACACAGGAAACAGCTATGACCATGATTACGCCAAGCTGAATGACGATTAACAAAGTAAAGAACAA |
| pSG1-compRPN4_rev | CCATTAACTTTAAACCATCTTCGACCGTCATGTCCTTTCTAAGTAGAGTACAAAGTATCGCGAAAATAC |
| New compSUT1 1480bp up | ttcacacaggaaacagctatgaccatgattacgccaagctGAAGATTTTTATTGTCCCCTTTGTTTCA |
| pSG1-comp19.4342_rev | CCATTAACTTTAAACCATCTTCGACCGTCATGTCCTTTCTCGAACAAGCTTAAAATGTTCCAGTTCCAGCTG |
| Comp_ROB1_For | TTCACACAGGAAACAGCTATGACCATGATTACGCCAAGCTTGACATTCAAGCCACAGCTC |
| pSG1-compROB1_rev | CCATTAACTTTAAACCATCTTCGACCGTCATGTCCTTTCTCACCCTTTAGTCGGTGGTGGAAATGCACGT |
| EFG1 comp F | TTC ACA CAG GAA ACA GCT ATG ACC ATG ATT ACG CCA AGC TAT TTC AAT CTT ATT TAA CTA TCC |
| EFG1 comp R | CCA TTA ACT TTA AAC CAT CTT CGA CCG TCA TGT CCT TTC TAT ATT ATT ATT ATT ACA CAC TTG |
| EFG1 comp F2 | TTC ACA CAG GAA ACA GCT ATG ACC ATG ATT ACG CCA AGC TGT AAT TTC AAT CTT ATT TAA CTA TCC AAT TAT CCC AAC |
| EFG1 comp R2 | CCA TTA ACT TTA AAC CAT CTT CGA CCG TCA TGT CCT TTC TCC CAT GTA TAT GCA TAT TTG TAC CTT CCG CAT TAG ACG C |
| HAP3-F | CAT CAC GGA GAC GAT TCT CA |
| HAP3-R | GGG ATT CGT TGG TAA TGG TG |
| RPN4-F | TCA CCT TCA GCC ATT TCT CC |
| RPN4-R | TCG TCG TCG TCA ACT GAT TC |
| ZAP1-F | CGG GTT CAT TCA GGA GAA AA |
| ZAP1-R | ACG ATT GTC CAC ATG CTT CA |
| rim101-F | AGT CCA TGT CCC ATT GAA GC |
| rim101-R | TGC ATT CAT CGA GTT TGC TC |
| hap43-F | AAG CAA AAC CAA TGC CTG AC |
| hap43-R | TGA TTG GTG AGG GTT GTT CA |
| orf19.4342-F | TTA GCC ACT CCT CCA CCT TC |
| orf19.4342-R | GGT TGT TGT GGT TGT GGT TG |
| rob1-F | GAG GAA TTT TAC GCC CAA CA |
| rob1-R | TCT TGT GGT TGT GGT TCG TC |
| ALS3-F | CCACTTCACAATCCCCATC |
| ALS3-R | CAGCAGTAGTAGTAACAGTAGTAGTTTCATC |
| DDR48-F | CACTGACAGTTACGGTTCTTCC |
| DDR48-R | AATAGTCAGAAGATCCATAGGAGTC |
| SAP6-F | GAACATTAAGTGTCGGTTTAAG |
| SAP6-R | AATCGTAAACAATGTAAGCTG |
| HWP1-F | CACTACATTCTGTCCATTGAC |
| HWP1-R | GCTGGAGTCATTTCAGGAAC |
| RBT5-F | GCCGCTGAAACCACCAAGG |
| RBT5-R | GGAAACAGAAGCAACGTCTG |
| TDH3-F | ATCCCACAAGGACTGGAGA |
| TDH3-R | GCAGAAGCTTTAGCAACGTG |
| ZAP1-OE-F | GGT CGA ATC CCA TTT CGG GTG CTC ACA CTA CCA GGT TAC GAC TAA TGC TAT GAC TGC TCC CCT TTT AGA AAA GTT TGA AAA TAT TAA CTT CTT GAT GCA ATA TTG ATC AAG CTT GCC TCG TCC CC |
| ZAP1-OE-R | AAA GCA ATC ATC AAG ATC ATT GCA AAA TAC GTC TTT GCA AAA ATC CAA GTC GTC CAA CGC AGG ACA TGA AAA GTT GAA ATT GGG TTG ATT AGA AAT AGG TTC CAT TGT TAA TTA ATT TGA TTG TAA AGT TTG TTG ATG |
| ZRT2-OE-F | CAC TGT TTT TCT GTC TTG TTG TTC CAA ATA ACC ACT AAT ATT TCT CTT ATA CTT GAC GAT TTT TGG TGA CCT ATT ATA GCT GGC AAG TGA AAG TGA ATT AAT AAT ATC AAG CTT GCC TCG TCC CC |
| ZRT2-OE-R | GGA GGA AAT TCT TGT TCC AAT ATT CCC ATT ATA ATC ATT ATC TGT GGG ACA TTC ATC CCT TTT GTT CAA ATA TTC AAA TAC TTG GGA AAT GGA ATC ACT ATT CAT TGT TAA TTA ATT TGA TTG TAA AGT TTG TTG ATG |
